# 3D Osteocyte Networks under Pulsatile Unidirectional Fluid Flow Stimuli (PUFFS)

**DOI:** 10.1021/acsbiomaterials.5c00730

**Published:** 2025-10-02

**Authors:** Anna-Blessing Merife, Arun Poudel, Angelika Polshikova, Zachary J. Geffert, Jason A. Horton, Mohammad Mehedi Hasan Akash, Anupam Pandey, Saikat Basu, Daniel Fougnier, Pranav Soman

**Affiliations:** † Department of Chemical and Biomedical Engineering, L.C. Smith College of Engineering Syracuse University, Syracuse, New York 13244, United States; ‡ Department of Neuroscience and Physiology, Alan and Marlene Norton College of Medicine, 12302 SUNY Upstate Medical University, Syracuse, New York 13210, United States; § Department of Mechanical Engineering, 2019 South Dakota State University, Brookings, South Dakota 57007, United States; ∥ Department of Mechanical and Aerospace Engineering, L.C. Smith College of Engineering Syracuse University, Syracuse, New York 13244, United States

**Keywords:** osteocytes, 3D cultures, MLO-Y4, mechanical stimuli, in vitro model, microfluidic, chip

## Abstract

Although osteocytes are known to play a key role in skeletal mechanoadaptation, few in vitro models have investigated how pulsatile mechanical stimuli influence the properties of three-dimensional (3D) osteocyte networks. Here, we design and develop a microfluidic-based in vitro model to study 3D osteocyte networks cultured under Pulsatile Unidirectional Fluid Flow Stimuli (PUFFS). Digital light projection stereolithography was used to design and fabricate a three-chambered polydimethylsiloxane (PDMS) microfluidic chip. Model osteocytes (murine MLO-Y4) were encapsulated in the collagen matrix within the chip to form self-assembled three-dimensional (3D) cell networks. Daily stimulus in the form of PUFFS was then applied for up to 21 days. A combination of experiments, computational simulation, and analytical modeling was used to characterize the mechanical environment experienced by embedded cells during PUFFS. Viability, morphology, cell-connectivity, expression of key proteins, gene expression, and real-time calcium signaling within 3D osteocyte networks were characterized at select time points and compared to static conditions. Results show that PUFFS stimulation at 0.33 and 1.66 Hz can initiate mechanotransduction via calcium signals that are propagated across the network of collagen-encapsulated osteocytes via the Cx43 junctions. Furthermore, osteocytes cultured in these devices maintain expression of several key osteocyte genes for up to 21 days. Taken together, this model can potentially serve as a testbed to study how 3D osteocyte networks respond to dynamic mechanical stimulation relevant to skeletal tissues.

## Graphical abstract

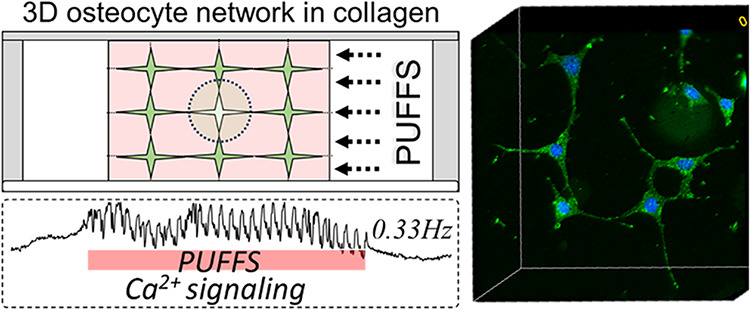

## Introduction

Osteocytes are the primary mechanosensory cells within bone tissue. Mechanical loading creates an interstitial fluid flow that induces dynamic signaling across three-dimensional (3D) networks of interconnected osteocytes. In turn, these signals spatially coordinate osteoblastic bone deposition and osteoclastic resorption at the bone surface via paracrine and juxtacrine factors.^1–9^ Mechanical stimulation is necessary for osteocyte function,^10^ and disruption of their mechanotransduction is implicated in many skeletal disorders.^11–16^ Contemporary in vivo and ex vivo models have yet to reveal the mechanisms that propagate short-term signals such as calcium across 3D networks, which modulate long-term remodeling responses.^11–22^ This is largely due to the dependence on live animals or human explant tissue,^23–26^ which requires expensive and complicated experimental apparatus^27–29^ with low throughput, poor reproducibility, and superficial depth of observation.

To address these challenges, many complementary in vitro models have been developed, although most studies continue to rely on simpler two-dimensional (2D) designs that do not replicate the complex 3D architecture and dynamic signaling of osteocyte networks in vivo. For example, bulk stimulation of osteocyte monolayers via flow chambers subjects nearly all cells in the system to identical and simultaneous stimuli.^30–32^ More sophisticated tools such as nanoindentation can stimulate individual osteocytes within patterned 2D networks^33–37^ yet this does not consider the 3D microenvironment. Transwell models and microfluidic devices^38–48^ have been used to study paracrine signaling in a coculture setup; however, applying regionally confined mechanical stimulation, especially during long-term cultures, remains challenging. Pseudo-3D network models have also been developed by culturing osteocytes on the surfaces of 3D microbeads^49^ or embedding cells within mineralized 3D constructs to mimic in vivo microenvironmental conditions;^48^ however, their opacity precludes real-time visualization of signaling behavior. As a result, cells encapsulated within collagen continue to be the gold standard for studying real-time signaling within 3D osteocyte networks.^49–60^ However, new models need to be developed that allow (i) 3D osteocyte culture, (ii) application of defined mechanical stimuli, (iii) longitudinal study of real-time signal propagation between interconnected cells, and (iv) long-term changes in osteocyte morphology, viability, proliferation, and gene expression.

In this work, we report on the design, development, and characterization of a new experimental model that combines each of these features in a microfluidic platform. This model uses osteocytic MLO-Y4 cells suspended in a 3D collagen extracellular matrix. Within this matrix, individual osteocytes self-assemble into networks of interconnected cells that propagate signals via gap junctions and other mechanisms and maintain this organization for at least 3 weeks. The three-channel design of our microfluidic system affords high-resolution live cell imaging of Ca^2+^ signaling dynamics and potentially other signals with appropriate reporters and is amenable to subsequent immunofluorescence studies of fixed devices to examine cell morphology and protein expression. We developed an apparatus to apply Pulsed Unidirectional Fluid Flow Stimuli (PUFFS) to simultaneously apply physiological levels of both fluid-shear stimulation (modeling the interstitial fluid flow induced upon bone loading) and cyclic compression–relaxation of the extracellular matrix (modeling cellular responses to matrix deformation) that occur during loading. The PUFFS apparatus is compatible with fluorescence microscopy, enabling the real-time visualization of Ca^2+^ signal initiation and propagation across the network of interconnected osteocytes upon stimulation. Using a combination of empirical experiments and in silico modeling and simulation approaches, we characterized the mechanical microenvironment experienced by osteocytes in 3D networks during PUFFS. Lastly, we demonstrate that PUFFS can be applied to the 3D osteocyte networks for at least 21 days, allowing long-term assessment of changes in cell viability, morphology, gene expression, and real-time signaling dynamics in response to stimulation.

## Results and Discussion

### Design and Fabrication of Multichambered Microfluidic Chips

For a long-term culture of 3D osteocyte networks, we designed and developed three-chambered microfluidic chips in PDMS (Figure 1A). Briefly, digital light projection (DLP) stereolithography was used to print a negative master mold using polyethylene glycol diacrylate (PEGDA) resin, followed by replica-casting using PDMS and irreversibly bonding the PDMS molds to glass coverslips (22 mm × 22 mm, Figure 1B); the details of this process are explained in the Materials and Methods section. The final devices consist of three chambers with inlet and outlet ports (2 mm diameter) and a central chamber (850 μm wide, to house osteocyte-laden collagen) flanked on either side by two chambers (∼500 μm wide, for PUFFS and media exchange), separated by an array of posts with an interpost gap of 65 μm (Figure 1Bi). The height of all the chambers within the chip was 250 μm. Post fabrication, the chips were surface-coated with polydopamine (PDA), and MLO-Y4s in type I collagen (2.5 mg/mL)^61^ at a final concentration of 1 × 10^5^ cells/mL were thermally cross-linked (37 °C, 30 min) within the central chamber of the chip (Ch#2). The post array prevents leakage of cell solution into side chambers during the gelation of collagen in chamber 2. For dynamic conditions, PUFFS (0.33, 15 min/daily; from Day 3 to 21) were applied to chamber 3 (Ch#3) of the chips, while for static conditions, no stimuli were applied (Figure 1Bii).

**Figure 1:**
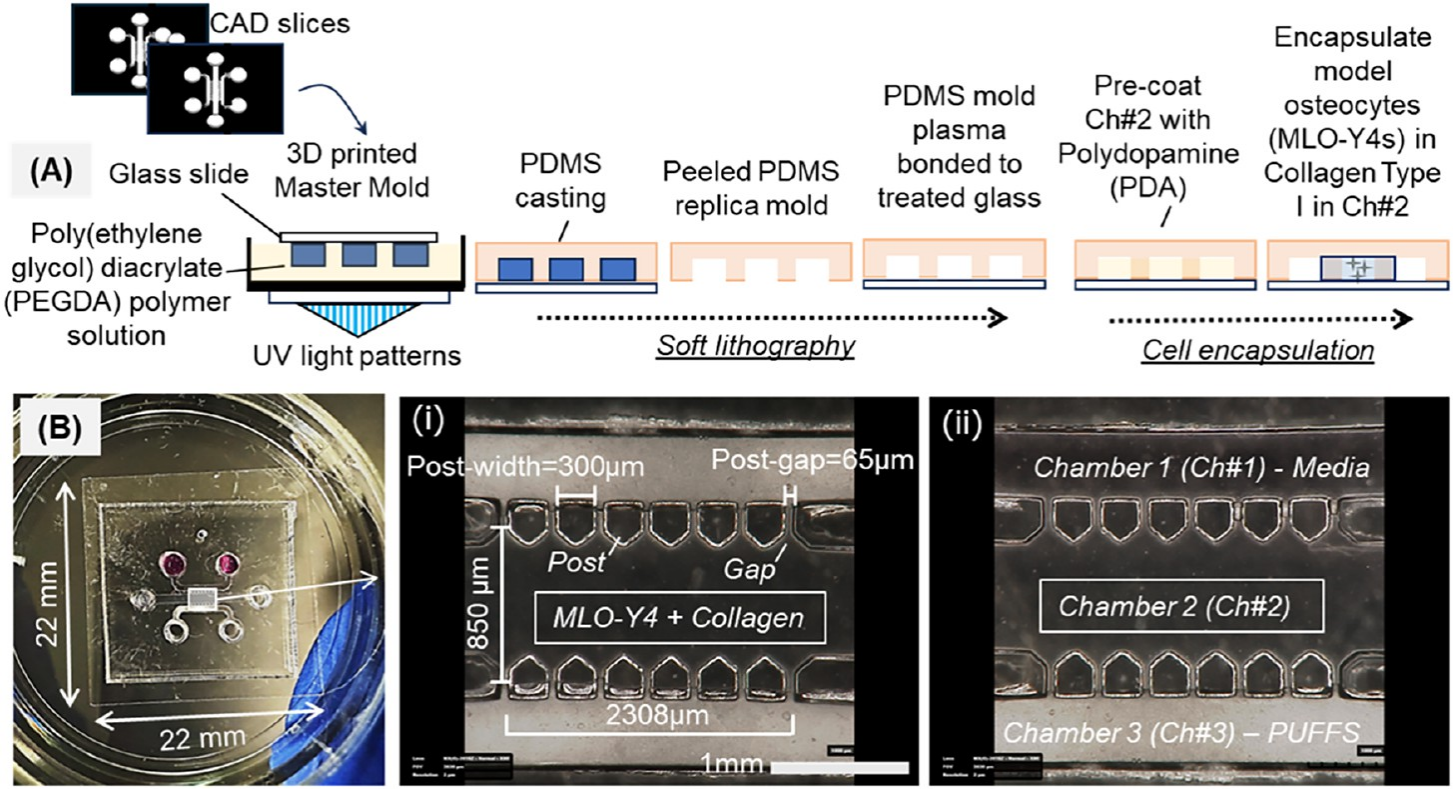
(A) Fabrication process flow to develop PDMS microfluidic chips with an MLO-Y4-laden collagen barrier in Ch#2. (B) Representative picture of a three-chambered chip showing three inlet–outlet pairs. (i), (ii) Pictures show relevant dimensions of chambers 1, 2, and 3 (Ch#1, #2, #3). Ch#2 house MLO-Y4 + collagen gel. For a dynamic culture, PUFFS are applied in Ch#3 with a static media culture in Ch#1. For static culture, media are present in both Ch#1 and Ch#3.

### Setup Design, Development, and Optimization for PUFFS

To generate cyclic mechanical stimuli, we designed a new experimental setup that includes a peristaltic pump, a controller, and connector tubings to generate pulsed unidirectional fluid flow stimuli or PUFFS applied at a frequency setting of 0.33 Hz (or 1.66 Hz) in chamber #3 of the microfluidic chips. A pump pressure of 30 kPa results in a velocity of 0.018 m/s in chamber #3 of the chip; these experimental conditions do not cause any disruptions to the cross-linked collagen barrier for the duration of the study. Figure 2A shows the setup used to apply PUFFS to 3 independent chips. Next, we tested the ability of this setup to reliably capture calcium responses of 3D osteocyte culture during PUFFS. After gelation of MLO-Y4-laden collagen in chamber 2 of the chips, Fluo-4 AM calcium dye was incubated and washed, and PUFFS were applied in Ch#3 for 60 s. The first setup coined as “open-loop” involved an unidirectional flow with the outlet tubing being open. Representative plots of calcium signaling in individual MLO-Y4s during PUFFS showed an unstable profile; this is potentially due to the negative pressure and associated flow fluctuations due to backflows (Figure 2Ci). During open-loop perfusion experiments, the inlet port of chamber 3 is connected to a tubing, while the outlet port is exposed to atmospheric conditions. Due to this, media continue to accumulate near the outlet port, and this results in sinusoidal wave-like fluctuations even after addition of a stabilizer (Figure 2Cii). To improve this setup, we tested a “closed-loop” setup, where both inlet and outlet tubings were used to generate a recycled unidirectional flow. This resulted in more stable signals, but slight movement of the chip during the application of PUFFS caused fluctuations in the signal. To further improve the reproducibility of signals, a stabilizer was designed and 3D-printed to mitigate unwarranted movement from the connected chips during imaging; details of the stabilizer are provided in Figures 2B and S1. Since the use of the stabilizer and running PUFFS under closed-loop conditions provided reproducible calcium signal recordings, this setup was used for all experiments in this work. We do not anticipate that recycling media will introduce any biochemical artifacts, as the duration of PUFFS is short (15 min every 24 h), and the media are changed after each PUFFS experiment for the entire duration of the experiment (21 days).

**Figure 2:**
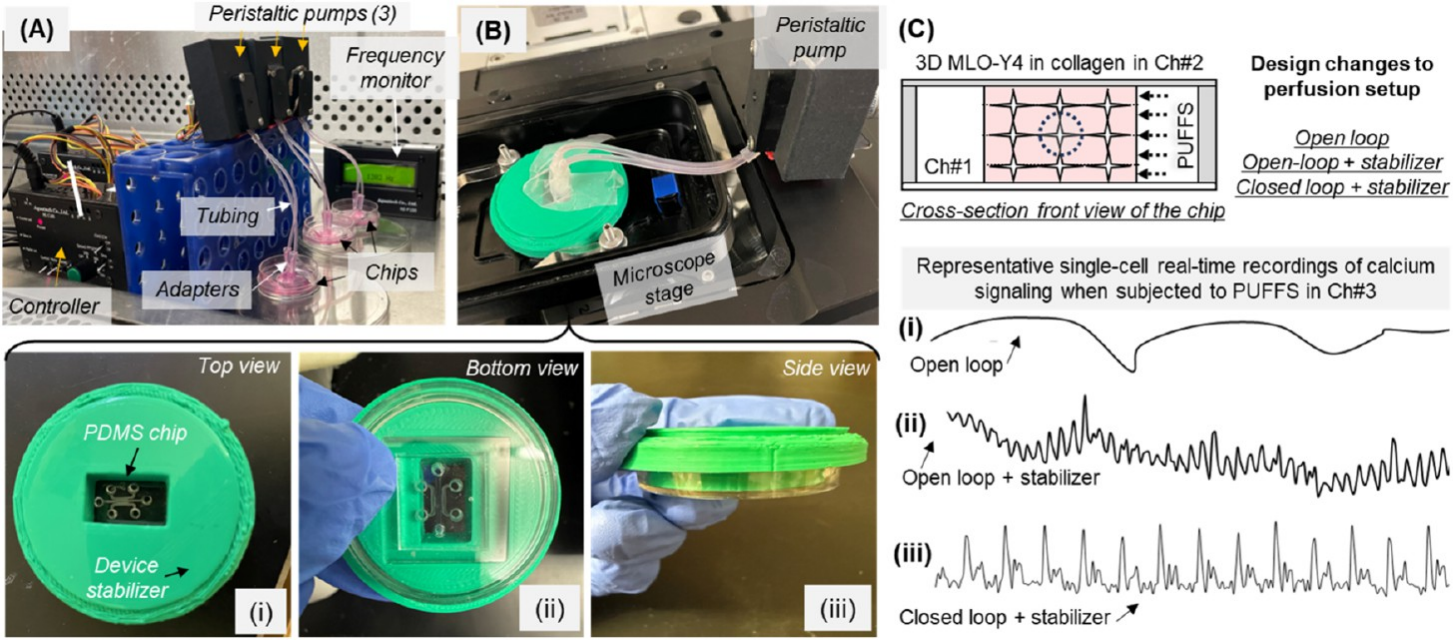
(A) Picture of the setup showing parallel application of PUFFS to 3 chips. (B) Device stabilizer (shown in green) fitted onto the microscope stage holds the chips and enables reproducible collection of real-time calcium signaling data during the application of PUFFS in Ch#3; (i–iii) various views of the assembly of the chip within the stabilizer. (C) Schematic showing the cross-sectional view of the chip; (i–iii) changes in single-cell calcium signaling during PUFFS under different setup configurations.

### Characterization of the Mechanical Microenvironment Experienced by Cells during PUFFS

Before the biological characterization of osteocyte-laden collagen, it was important to understand the stresses experienced within the collagen gel during PUFFS. For the collagen concentration used in this work (2.5 mg/mL), the storage and loss moduli were calculated as 159 ± 33.51 and 53.33 ± 9.6 Pa, respectively (Figure S2). To characterize the stress and velocity profiles in cross-linked collagen when subjected to PUFFS, we used a combination of experiments, simulation, and modeling. First, collagen solution (2.5 mg/mL) was mixed with fluorescent beads (1 μm diameter) and cross-linked within chamber #2 of the chip. Upon application of PUFFS (0.33 Hz), the collagen gel compresses and relaxes with each fluid flow pulse (Figure 3Ai,ii and Movie S1). Although beads encapsulated within collagen aggregate into clusters (∼25 μm), their displacements in various regions within collagen was used to generate a vector field map; here the length of the blue arrow indicated the magnitude of displacement (Figure 3Aiii). Results show a bead displacement of 46.8 μm in the collagen subregion that is proximal to PUFFS and 32.5 μm in those that are distal to PUFFS. Based on chip dimensions, we calculated a maximum shear stress of 56.17 kPa at the collagen surface (interface of Ch#2 and Ch#3). To calculate the stress within collagen located in chamber 2 (Ch#2), we developed an elasticity model (Figure 2Bi,ii), where we approximate the shear stress as normal loading (*p*_*c*_ in Figure 3Ci acting perpendicular to the collagen surface in Ch#3). This approximation is motivated by the observation that the measured displacement in the collagen layer is predominantly in the direction normal to the interface. In this model, we assume the collagen layer to be purely elastic, and normal loading between two adjacent posts with the layer thickness (2 h) represents the width of collagen in chamber 2. Figure 3Biii shows the distribution of compressive stress in collagen (σ_*yy*_) during PUFFS. Note that, during PUFFS, the stress is maximum in chamber 3 (collagen region between two adjacent microposts), reaching a value of 1 (blue color in Figure 3Cii), while regions behind the posts remain relatively stress-free (marked by the red color in Figure 3Cii). This is the reason we choose to analyze only the calcium signaling of MLO-Y4s located between the posts in this study—the region that experiences stresses during PUFFS. Three cross sections marked by red dashed lines in Figure 3Bii were used to assess how the compressive stress (σyy) varies in collagen subregions proximal, central, and distal to PUFFS. Taking the highest value of 56.17 kPa (*p*_*c*_) experienced by the collagen at the interface of Ch#3 and Ch#2, subregions proximal to PUFFS (0–285 μm) will experience a stress range of 56.17–45.16 kPa, the central subregion (286–570 μm) will experience a stress range of 45.16–22.47 kPa, while the distal subregion (571–855 μm) will experience a stress range of 22.47–11.23 kPa (Figure 3Biv). Lastly, to simulate the velocity distribution within the collagen gel during PUFFS, we developed an Eulerian viscous two-phase model, treating water as the primary phase (Ch no. 3) and the collagen gel as the secondary phase (Ch#2; Figure 3Bv). The velocity contour plot in the collagen bulk reveals a high-velocity region at the collagen interface between Ch#3 and Ch#2 and at the edges of the posts and a decline in velocity within the bulk (Ch#2) due to collagen’s viscous resistance. By comparing the experimentally measured bead velocities at specific points within the collagen to the simulated values, we identified correction factors that aligned the numerical predictions with the experimental data, thereby validating our simulation model. Results show that the spatial demarcations of the simulated flow barriers in the collagen (Ch#2) match well with the experimental trends. Details related to experimental calculations, analytical modeling, and simulations can be found in the Supporting Section (Figures S3–S9 and Table S1).

**Figure 3:**
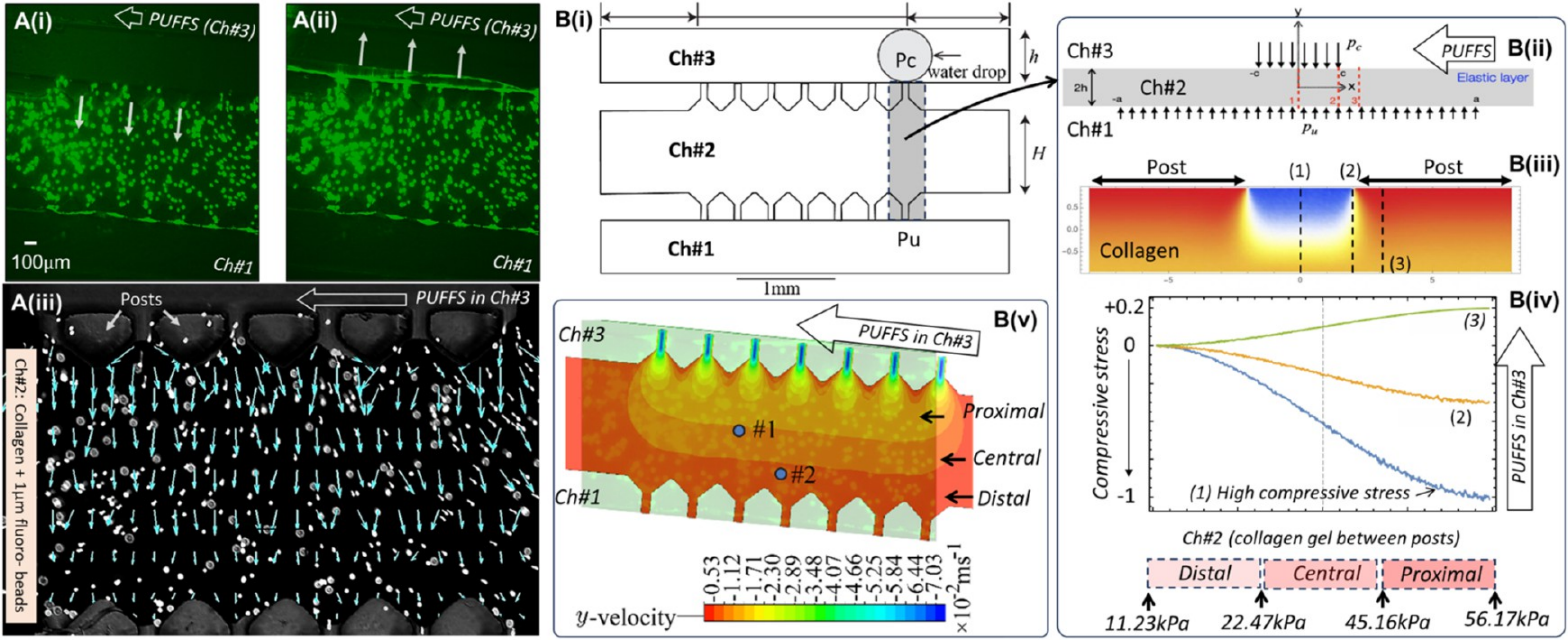
(A (i), (ii)) Fluorescence images showing the movement of bead-laden collagen during PUFFS. (A (iii)) Vector map superimposed on a bright-field image showing the magnitude of bead displacement. (Bi) Simplified geometry of the chip, (B (ii)–(iv)) Elastic modeling of collagen between two adjacent posts. (B (v)) Velocity distribution within collagen during PUFFS. Scale bar = 100 μm.

### Viability, Morphology, and Connectivity of 3D Osteocyte Networks Subjected to PUFFS

Chips with MLO-Y4 osteocytes encapsulated within collagen were subjected to PUFFS, and their viability was assessed using a live and dead staining assay (Figure 4A). Results show a decrease in viability for both static (no PUFFS applied) and dynamic (PUFFS applied from Day 3 to 21, 0.33 Hz) conditions. For instance, Figure 4B shows that viability decreased from 0.94 ± 0.034 on Day 3 to 0.81 ± 0.098 on Day 7 to 0.69 ± 0.065 by Day 21 under static conditions. Under dynamic conditions, viability decreased from 0.93 ± 0.016 on Day 3 to 0.88 ± 0.018 on Day 7 to 0.81 ± 0.086 by Day 21. We interpret the gradual decline in viability, more specifically a gradual accumulation of the proportion of cells staining with propidium iodide, to be the result of gradual but normal otherwise cell death, resulting in the accumulation of the nuclear material that is “trapped” in the collagen hydrogel, not rapidly cleared by degradation or immune cell activity. At least 3 independent chips (samples) were used for this study, and images were taken from the entire region in chamber #2.

**Figure 4:**
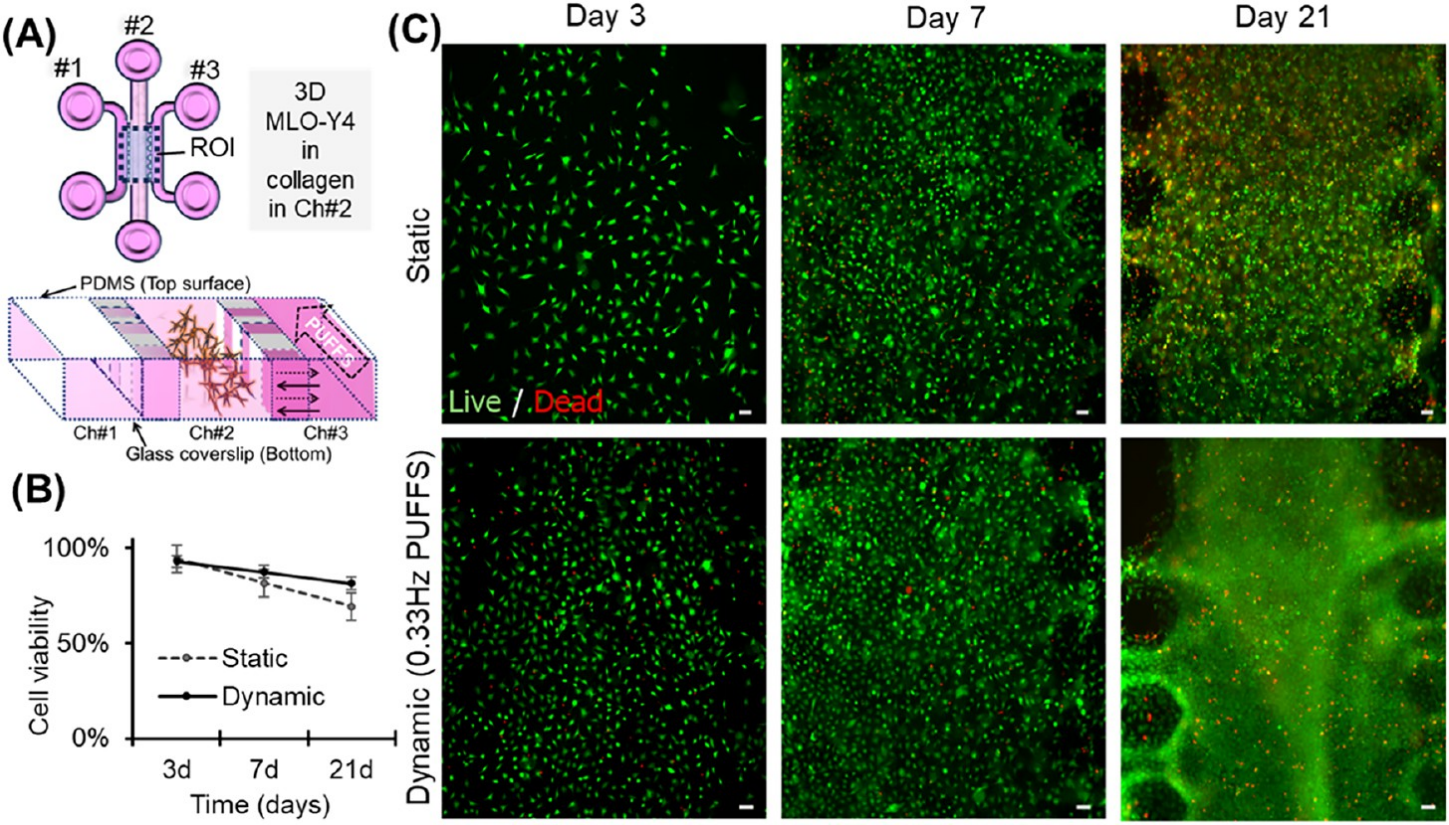
(A) Schematic of the top view and cross-sectional view of the chip. (B) Cell viability as a function of culture duration under static and dynamic (PUFFS, 0.33 Hz) conditions. (C) Representative fluorescence microscopy images captured from chamber 2 showing live (green) and dead (red) cells. Scale bar = 50 μm.

MLO-Y4 morphology was assessed by staining cells for the nucleus (blue) and f-actin (green; Figure 5A–D). Since the total height of the collagen was ∼250 μm, we took images at different z-depths from the bottom glass slide. Images taken from ∼70 μm from the bottom were denoted by (i) in Figure 5A–D, and images taken from a z-plane close to the top PDMS surface (∼200 μm from the bottom) were denoted by (ii) in Figure 5A–D. On Day 3, we observed many cells on the bottom, some cells in the middle section (i), but no cells in the top plane (ii). This is reasoned to be the result of the gravity-induced setting of cells during collagen gelation. With longer culture durations, gradual expansion of cell number allowed the cells to populate the entire depth of the collagen with highly spread-out cells in all planes on Day 21, achieving a density exceeding that reported in the lamellar bone formed during endochondral fracture repair in rats.^62^ Cell nuclei from captured images and from three independent chips were used to calculate the number of cells per unit area (Figure 5D). Results show an increase in cell number with culture duration under both static and dynamic conditions. Under static conditions, cell number increased from 139 ± 92 (Day 3) to 591 ± 102 (Day 7) to 2482 ± 596 (Day 21), while under PUFFS conditions, cell number increased from 211 ± 3 (Day 3) to 587 ± 352 (Day 7) to 2743 ± 848 (Day 21). We found it challenging to identify connections between cells encapsulated within a 3D collagen matrix, especially at later time points when cells are closely packed. Also, since we wanted to visualize the entire region of osteocyte-laden collagen in chamber 2, a 10× objective was used for imaging, which makes it difficult to visualize the connections between adjacent osteocytes. A representative z-stack movie file (Movie S3 in the SI) clearly shows that osteocytes form a connection within the 3D collagen matrix by Day 3. Therefore, we use cell–nuclei separation distances as a criterion to generate 3D cell connectivity maps. Higher resolution images taken from Day 3 were analyzed using ImageJ (FIJI; Figure S12) to calculate the distance between neighboring cells using the Gaussian distance formula. Based on this, the nuclei separation distances ≤ 50um were assumed to be connected cell pairs (solid lines), while individual nuclei are represented as red dots (Figure 5E). The 3D maps provide a visual representation that shows an overall increase in cell number and cell migration into the 3D collagen matrix, and cell connectivity with longer culture durations. Maps show some cell aggregation in the dynamic condition compared to the static condition, which aligns well with observed cell morphology.

**Figure 5:**
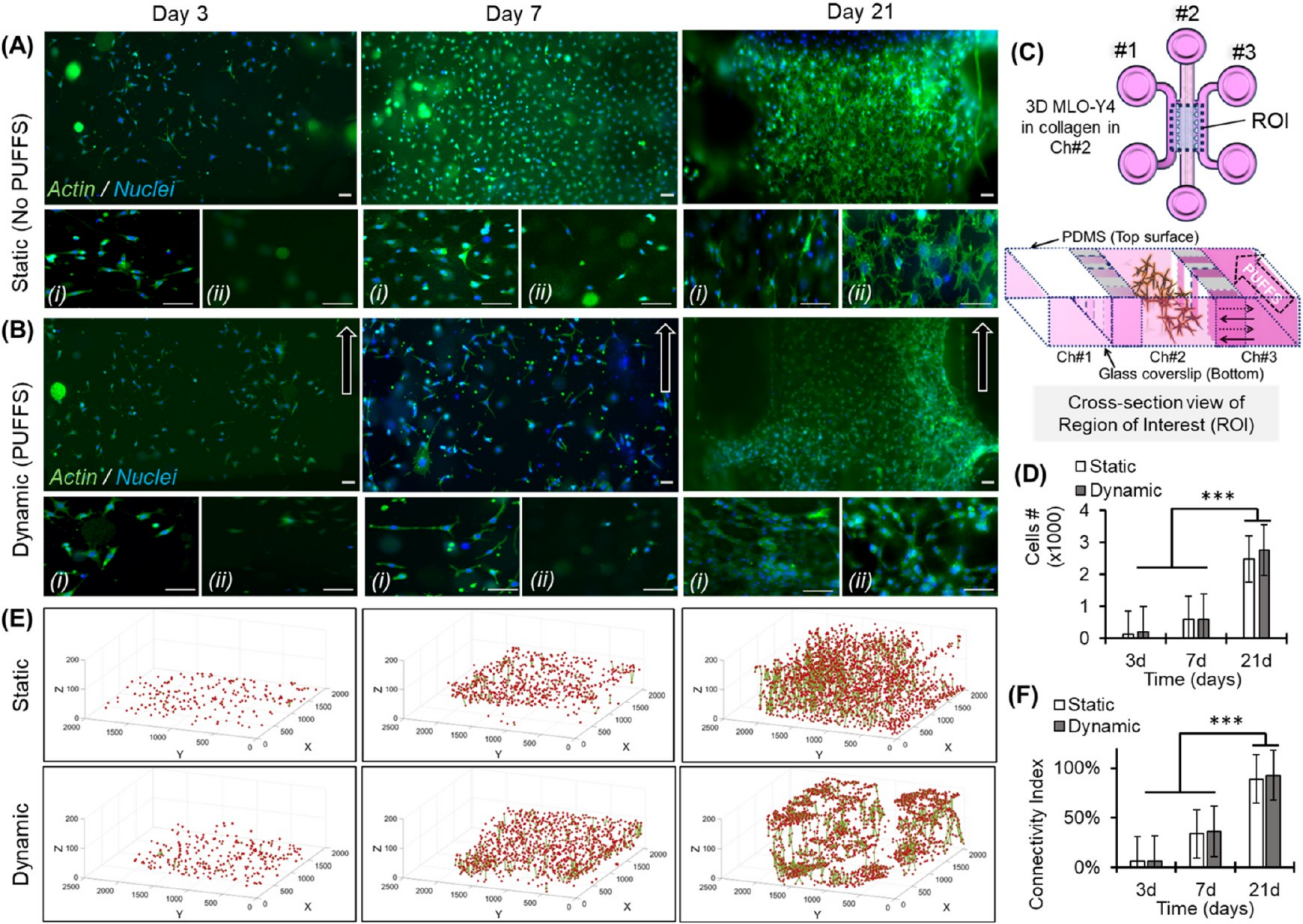
(A, B) Representative image of MLO-Y4 morphology in static and dynamic conditions; (i, ii) images taken at ∼70 and ∼200 μm from the bottom glass coverslip. The white arrow indicates the direction of PUFFS. (C) Schematic of the chip, (D) plot showing cell number per unit area on Days 3, 7, and 21 (*p* < 0.001). (E) 3D cell-connectivity maps; red dot = individual cell nuclei, green line = connection with adjacent cells. (F) Connectivity index on Days 3, 7, and 21. Scale bar = 50 μm. *** *p* ≤ 0.0010.

### Changes in Gene Expression in 3D MLO-Y4 Networks under PUFFS

To assess the impact of PUFFS on gene expression by osteocytes, chips with 3D MLO-Y4-laden collagen gels were subjected to dynamic or static conditions for 7 or 21 days. The gene expression profiles of 10 mechanosensitive target genes (*Alpl*, *Gja1*, *Pdpn*, *Sost*, *Tnfsf11*, *Tnfsfr11b*, *Phex*, *Mepe*, *Dmp1*, and *Fgf23*; Tables S2 and S3) were analyzed via RT-qPCR, normalized to housekeeping genes (*Gapdh* and *Hsp90ab1*), and assessed via two-way ANOVA to assess statistical significance of differences between treatment groups as well as isolate the contributions of time (7 days/21 days), stimulus (Static/PUFFs), and the interaction of these terms (Figure 6). Alkaline phosphatase (*Alpl*) is expressed by osteoblasts and early osteocytes and facilitates the deposition of a mineralized matrix by hydrolyzing extracellular inorganic pyrophosphate, making it available for the formation of calcium hydroxyapatite. However, as cells transition from the osteoblast to osteocyte phenotype and become entombed in their mineralized matrix, ALPL expression and de novo mineralization decrease as part of the normal differentiation program, particularly when mechanically loaded.^63,64^ *Alpl* exhibited significant variance from time (10.1%), stimulus (40.0%), and interaction (26.7%, all *p* < 0.0001). Relative to 7-day static controls, Alpl was downregulated in cells exposed to PUFFS for 7 days (−3.73-fold, *p* = 0.0049) with pronounced downregulation at 21 days under both static (−26.3-fold, *p* < 0.0001) and PUFFS (−6.48-fold, *p* < 0.0001) conditions. There was no significant difference in Alpl expression in between cells exposed to PUFFS for 7 or 21 days (−1.74-fold, *p* = 0.1837).^65^ Downregulation of *Alpl* expression under PUFFS aligns with ERK1/2-mediated suppression of Runx2-driven expression under shear stress, reflecting cellular maturation rather than pathological responses. Our result for *Alpl* is consistent with osteoblasts reducing matrix mineralizing activity as they enter the early-to-intermediate stages of osteocytic differentiation; thus, the MLO-Y4 cell line is well-suited to the model.^61^

**Figure 6:**
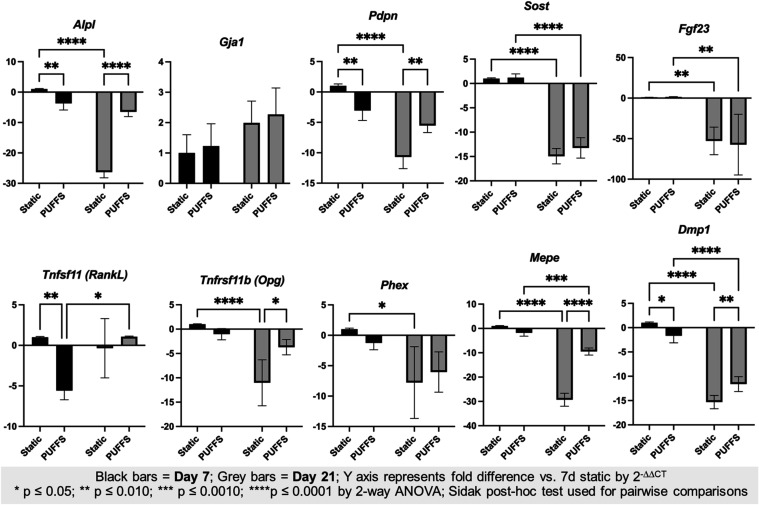
Gene expression by RT-qPCR for 10 osteocyte genes. Data shown are mean ± SD of *n* = 3–5 replicates, and brackets show statistically significant differences (*p* < 0.05) in gene expression between treatment groups by 2-way ANOVA (time*treatment). * *p* ≤ 0.05; ** *p* ≤0.010; *** *p* ≤ 0.0010; **** *p* ≤ 0.0001.

The gap junction protein α 1 (*Gja1*) transcript encodes the protein connexin 43 (CX43), which is integral to osteocyte mechanotransductive function in that it enables transmission of small signaling molecules (e.g., Ca^2+^) between interconnected osteocytes.^66,67^ Gap junction protein α 1 (Gja1/Cx43) showed no significant differences, suggesting that baseline gap junction integrity is transcriptionally stable, as indicated by immunofluorescence. Podoplanin (*Pdpn*), also known as protein E11, is implicated in the initial stages of osteocyte differentiation, is essential for the formation of dendritic processes, and has been suggested to act as a sensor for bone damage.^68,69^ Expression of *Pdpn* was suppressed by PUFFS (−3.06-fold, *p* = 0.0070 at 7 days; −5.52-fold at 21 days, *p* < 0.0010). Relative to 7-day static controls, downregulated *Pdpn* (−10.69-fold, *p* < 0.0001) was observed at 21 days. There was no significant difference in *Pdpn* expression between cultures exposed to PUFFS for 7 and 21 days (*p* = 0.1879). These changes appeared to be driven by the stimulus (50.1%) and interaction of time and stimulus terms (21.3%, *p* < 0.0001). This contrasts with research demonstrating that *Pdpn* increases with in vivo loading, which enhances osteocyte connectivity in dendritogenesis. Reduced *Pdpn* as observed here may indicate excessive shear stress activating RhoA/ROCK pathways that trigger cytoskeletal retraction of dendritic processes.^70^ Alternatively, this reduction of *Pdpn* with PUFFS may indicate a matrix-driven feedback mechanism because collagen gel stiffness may be insufficient to support dendrite extension, despite mechanical stimulation.^64^ Unloading of bone promotes the secretion of Sclerostin, encoded by the *Sost* gene, by osteocytes acting as a negative regulator of bone formation by inhibiting the Wnt/β-catenin signaling pathway.^71–73^ Expression of (*Sost*) was predominantly influenced by the interaction of time and stimulus variables (89.05%, *p* < 0.0001). In comparison to 7-day static controls, exposure to PUFFS for 7 days did not significantly affect *Sost* expression (1.19-fold, *p* = 0.9994) but was downregulated relative to 7 days in both static (−14.94-fold, *p* < 0.0001) and PUFFS (−13.23-fold, *p* < 0.0001) conditions. In vivo loading suppresses *Sost* via Piezo1 activation.^74^ It is possible that the less stiff environment provided by the collagen gel limits prevented mechanosensitive Piezo1 activation, leaving time-dependent silencing dominant. While Sost downregulation should activate Wnt signaling, the absence of anabolic gene upregulation (e.g., Alpl and Dmp1) suggests that reduction of Sost may be compensated by Secreted Wnt antagonists (e.g., Dkk1) not assayed here^64,75^ or via mechanically activated signaling through the Wnt/Ca^2+^ or Wnt/PCP pathways.^76^ *Fibroblast growth factor 23* (*Fgf23*) is expressed by osteocytes at the most advanced stage of differentiation. Mechanical strain has been suggested to modulate the expression of *FGF23*, which acts on the kidney to regulate systemic phosphate and vitamin D metabolism.^77–79^ While *Fgf23* was detected under both static and PUFFS conditions after 7 days in the device, expression was not significantly different between treatments (+1.48-fold, *p* = 0.9998). In contrast, *Fgf23* was barely detectable in MLO-Y4 cultured for 21 days under either static or PUFFS conditions. Fibroblast growth factor 23 (Fgf23) became undetectable at 21 days, correlating with Phex downregulation (−7.77-fold static 21 days). Mechanical loading in vivo requires the Phex-mediated cleavage of MEPE for Fgf23 maintenance. Mineral-free collagen gels, as studied here, may impair feedback via the Phex–Fgf23–MEPE axis, mimicking osteocyte dedifferentiation and disrupted phosphate homeostasis.^80^

Receptor activators of nuclear factor κB ligand (*Rank-L* or *Tnfsf11*) and osteoprotegerin (*Opg* or *Tnfsfr11b*) are secreted by osteocytes to modulate osteoclast development and resorptive activity. While *Rank-L* promotes osteoclast differentiation and bone resorption, *Opg* acts as a decoy receptor that neutralizes *Rank-L* to fine-tune the osteoclastic component of mechanoadaptation.^81,82^ Variance of *Rank-L* expression stemmed from time (16.4%), stimulus (17.3%), and interaction (39.5%), with PUFFS reducing *Rank-L* at 7 days (−5.58-fold, *p* = 0.0052). Mechanical suppression of *Rank-L* mirrors in vivo loading’s antiresorptive effects via *Mepe* upregulation.^83^ Osteoprotegerin (Opg/Tnfsfr11b) increased 2.97-fold at 21 days PUFFS (*p* = 0.0164), driven by the stimulus (40.9%, *p* = 0.0009) and interaction (16.6%, *p* = 0.0077). Sustained OPG elevation aligns with the mechanical promotion of decoy receptor production to buffer *Rank-L*.^84^ Prolonged mechanical stimulation enhances OPG production, consistent with in vivo loading suppressing resorption.^83^ Pulsatile fluid flow in MLO-Y4 cells has been shown to increase MEPE, lowering RANKL/OPG ratios and inhibiting osteoclastogenesis.^83^ It is recognized that the expression of the *Tnfsf11* and *Tnfrsf11b* transcripts may not consistently reflect the levels of their respective secreted protein. Attempts to assay levels of the soluble proteins in effluent media by ELISA were not successful, most likely due to their low abundance, which did not exceed the lower limit of detection. Nonetheless, these results suggest that prolonged mechanical stimulation enhances OPG production at the transcript level, which is consistent with in vivo loading suppressing resorption, and thus warrants further investigation.

The phosphate-regulating neutral endopeptidase (*Phex*) gene encodes a zinc metalloendopeptidase expressed in osteocytes that is involved in bone mineralization and phosphate homeostasis via its influence on *FGF23* expression.^85,86^ In two-way analysis, only treatment contributed significantly to the observed variance (44.1%, *p* = 0.0040). *Phex* showed stimulus-driven downregulation (−7.77-fold static 21 days, *p* = 0.0110), implicating mineral-free collagen gels in disrupting the Phex–Fgf23 axis critical for phosphate regulation.^80^

Osteocytic expression of matrix extracellular phosphoglycoprotein (*Mepe*) is modulated by mechanical stress, suggesting its involvement in adaptation to mechanical loading. Time (10.1%), stimulus (50.2%), and interaction (17.9%) were significant. PUFFS reduced *Mepe* at 21 days (−7.03-fold vs static). In pairwise comparisons, *Mepe* was suppressed by PUFFS at 21 days (−7.03-fold, *p* < 0.0001), and *Mepe* expression was also significantly reduced between 7 and 21 days in both static (−29.3-fold, *p* < 0.0001) and PUFFS-treated cultures (−7.03-fold, *p* = 0.0006). This pattern contrasts with the induction of *Mepe* observed with mechanical loading in vivo. This paradox may reflect overload stress or absent mineral feedback. Similar to *Mepe*, expression of the *Dentin matrix protein 1* (*Dmp1*) transcript by osteocytes is upregulated in response to mechanical loading, though the Dmp1 protein is involved in both positive and negative regulation of matrix mineralization and is dependent upon post-translational modification and cleavage into fragments of varying functions.^86–88^ Furthermore, both *MEPE* and *DMP1* proteins are substrates of *PHEX*, whose proteolytic activity releases acidic serine aspartate-rich *MEPE*-associated motif (ASARM) peptides that bind hydroxyapatite and negatively regulate further matrix mineralization.^89,90^ Two-way analysis of data for *Dmp1* expression showed that while time did not contribute significantly to the observed variance (0.1%, *p* = 0.3980), stimulus (77.8%, *p* < 0.0001), and the interaction of time and stimulus (4.6%, *p* = 0.0002 were significant factors). There was no significant difference in *Mepe* expression between static and PUFFS-treated cells at 7 days (−1.83-fold, *p* = 0.1690); at 21 days, PUFFS significantly reduced expression (*p* < 0.0001) compared to 21-day static treatment. Dmp1 expression was reduced between 7 and 21 days in culture for both static (−15.31-fold, *p* < 0.0001) and PUFFS conditions (+3.09-fold, *p* < 0.0001). Dmp1 was downregulated by PUFFS (−5.17-fold at 21 days, *p* = 0.0039), opposing in vivo loading’s enhancer-driven Dmp1 activation,^80^ suggesting nonphysiological PUFFS parameters. The observed expression reductions of Mepe, Dmp1, and FGF23 are not consistent with the anticipated anabolic response. This pattern may reflect the limitations of the MLO-Y4 cell line to model the late and terminal stages of osteocyte differentiation when MEPE, DMP1, and FGF23 would be expected to reach peak expression and is further constrained by the absence of mineral-matrix feedback mechanisms necessary for full osteocytic gene expression.^64,91^ Studies by other investigators using a range of both 2D and 3D models with MLO-Y4, other cell lines, and primary cells and contrasted against in vivo experiments demonstrate that MEPE and DMP1 expression is highly dependent on matrix mineralization and shows complex temporal regulation under mechanical loading.^92,93^ In mineralized environments, these genes are upregulated by mechanical stimulation, but in nonmineralized collagen systems, their expression may be compromised despite mechanical stimulation.^63,94^

The application of dynamic mechanical stimulation (PUFFS) to MLO-Y4 osteocyte-like cells in a 3D collagen bioreactor system revealed the complex temporal and stimulus-dependent regulation of genes governing bone mineralization, osteocyte differentiation, and osteoclast-osteoblast coupling. Key patterns include (1) time-driven suppression of mineralization regulators (*Alpl*, *Mepe*, and *Dmp1*) independent of the mechanical stimulus,^84^ (2) PUFFS-mediated anticatabolic effects via RANKL/OPG modulation, (3) culture duration dominance over *Sost* expression, and (4) loss of mature osteocyte markers (*Fgf23*) at later time points. Despite *Sost* suppression, anabolic genes (*Alpl* and *Dmp1*) remain low. This mirrors β-catenin-independent Wnt signaling (e.g., Wnt/Ca^2+^) activated by mechanical stress, bypassing transcriptional targets like *Alpl*.^65^ Culture duration eclipses mechanical effects on Sost and Fgf23, highlighting limitations of prolonged in vitro osteocyte models. These findings underscore the need to optimize mechanical parameters (e.g., shear stress magnitude: 0.5–3 Pa);^74^ and incorporate mineral phases to better recapitulate the in vivo microenvironment of osteocytes. Mechanistically, PUFFS aligned with ERK1/2-mediated Runx2 suppression (Alpl) and RhoA/ROCK-driven dendrite retraction (*Pdpn)* but diverged in Wnt (*Sost*) and mineralization pathways, perhaps due to constraints of using a nonmineralized collagen gel.

In developing this model, we found the opacity of mineralized matrices (e.g., inclusion of granular hydroxyapatite confounded high-resolution 3D imaging, particularly in recording live calcium signaling responses), leading to our adoption of a collagen-only ECM environment. Nonetheless, we recognize this as a limitation of the physiological relevance of the current study. The absence of mineralization in our collagen model represents a fundamental limitation that significantly impacts the physiological relevance of our findings, particularly regarding Phex and Fgf23 gene expression.^95^ Matrix mineralization serves as a critical trigger for osteocyte maturation, with studies demonstrating that ″mineralization of the matrix surrounding the osteocyte is the trigger for cytodifferentiation from a plump immature form to a mature osteocyte″. Furthermore, they showed that in mineralized environments, osteocytes exhibit characteristic mature morphology and begin secreting sclerostin, whereas osteocytes in an unmineralized matrix remain in an immature state.

The Phex and Fgf23 genes are intimately connected through a mineralization-dependent regulatory network that is absent from our nonmineralized collagen environment. PHEX is a metalloendopeptidase that plays essential roles in phosphate homeostasis and bone mineralization, while FGF23 serves as a phosphaturic hormone whose expression is normally suppressed by functional PHEX and DMP1.^96^ Furthermore, the absence of hydroxyapatite prevents normal mineral-matrix feedback, compromising the PHEX–FGF23–MEPE axis, which is essential for phosphate regulation and matrix mineralization.^97^

While our model successfully demonstrates mechanotransduction capabilities and some aspects of osteocyte biology, the absence of mineralization severely limits its translational relevance for studying phosphate homeostasis, mineral metabolism, and mature osteocyte functions. The dramatic downregulation of Phex and loss of Fgf23 expression represent fundamental departures from physiological osteocyte behavior rather than responses to mechanical stimulation.^96^ The development of mineralized collagen systems would enable proper investigation of osteocyte responses to mechanical loading while also maintaining the optical accessibility, which makes our current platform valuable. Future iterations of our platform could incorporate monomolecular hydroxyapatite or other optically compatible calcium phosphate phases to restore normal Phex–Fgf23 axis function and responsiveness to systemic endocrine input.^98^

### Expression of Key Markers in 3D MLO-Y4 Osteocyte Networks

We stained the cells for key proteins related to osteocyte biology (Figure 7). Sclerostin (Sost), widely used to identify osteocytes, was stained for chips under both static and dynamic conditions on Days 7 and 21. Next, we stained for αvβ3 integrin, a receptor on osteocytes that facilitates attachment to collagen, and gap junction protein Cx43 that is known to facilitate mechanical stimuli-evoked calcium ion signaling. For both Cx43 and αvβ3, we observed that the staining was distributed over the entire cell surface, and higher levels of staining can be seen for the dynamic group as compared to the static control. Control experiments for all immunostains (exclusion of primary antibodies) show little to no nonspecific fluorescence signals (Figure S10).

**Figure 7:**
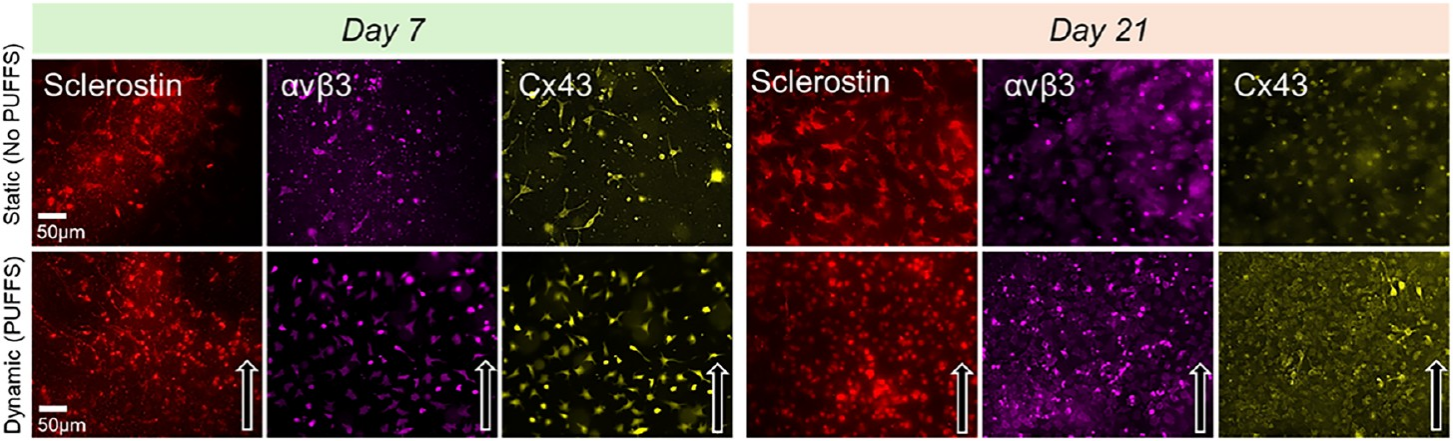
Representative fluorescence images showing the expression of sclerostin (red), connexin 43 (Cx43, yellow), and αvβ3 (pink) under static and dynamic conditions at Days 7 and 21 (scale bar = 50 μm). The white arrow indicates the direction of PUFFS.

### Real-Time Monitoring of Calcium Signaling within a 3D Osteocyte Network under PUFFS

Fluo-4 AM calcium staining in combination with time-lapse fluorescence microscopy was used to capture the changes in the calcium intensity of MLO-Y4-laden collagen in chamber #2. For every experiment, the baseline signal was captured under no stimulation conditions for ∼40 s, followed by the application of PUFFS (0.33 Hz) for ∼60 s in chamber #3. For every experiment, the average magnitude of baseline signals (0–40 s) was used to normalize the calcium signals. Representative fluorescence images and video captures of MLO-Y4 calcium signaling in chamber #2 show the propagation of the calcium signal across the MLO-Y4 network, starting from chamber #3 to chamber #1 (Figure 8A and Movie S2). We identified individual cells within the network and plotted changes in calcium intensity as a function of time (Figure 8B–C). We observed similar signals for both static and dynamic conditions. All MLO-Y4s showing oscillatory responses show one of the two signaling profiles (Figure 8C). One profile type returns to baseline fluorescence values after every PUFFS-induced calcium spike, while the other type exhibits a gradual increase in the overall fluorescence and does not come back to baseline signals even after PUFFS is stopped. We used *PeakFinder* (MATLAB) to assess the oscillatory frequency of cell-laden MLO-Y4s in chamber 2 (Figure 8D). We found that the frequency increases from 0.39 ± 0.83 Hz (Day 7) to 0.46 ± 0.22 Hz (Day 21) under static conditions. On the other hand, for chips subjected to daily PUFFS, the oscillation frequency decreases from 0.45 ± 0.04 Hz (Day 7) to 0.43 ± 0.33 (Day 21). We further characterized the number of MLO-Y4 cells showing oscillatory signals (Figure 8E). We found that, for static culture, MLO-Y4s exhibiting oscillatory response increased from ∼46% (Day 3) to ∼99% (Day 21), while for chips subjected to daily PUFFS (0.33 Hz), cells exhibiting oscillatory response increased from ∼70% (Day 3) to ∼83% (Day 21). Since both the mechanical deformation of the collagen matrix and cell-to-cell gap junction-based signaling could modulate their response, we repeated this experiment in the presence of a gap junction Cx43 blocker (GAP26). Briefly, on Days 7 and 21, the GAP26 solution is pipetted in side chambers for 45 min before applying PUFFS (0.33 Hz, 60 s) in chamber #3. Results show that, for static conditions, the total number of MLO-Y4s that exhibit an oscillatory response decreasing from 37% (Day 7) to 21% (Day 21), while for chips subjected to daily PUFFS (0.33 Hz), oscillatory cells decreased from 28% (Day 7) to 16% (Day 21). After blocking with GAP26 (Cx43 inhibitor), we saw an overall decrease in the number of cells showing the oscillatory response. This indicates that at early time points, when connectivity is low, inhibition of the gap junction does not play a significant role; this could mean that the responses we observe are more due to mechanical deformation. At longer culture durations (Day 21), with more cell-to-cell connectivity, there is a significant drop in the number of cells showing an oscillatory response, which indicates a significant role of gap junction-based signaling in addition to matrix deformation-induced signaling responses recorded during PUFFS.

**Figure 8:**
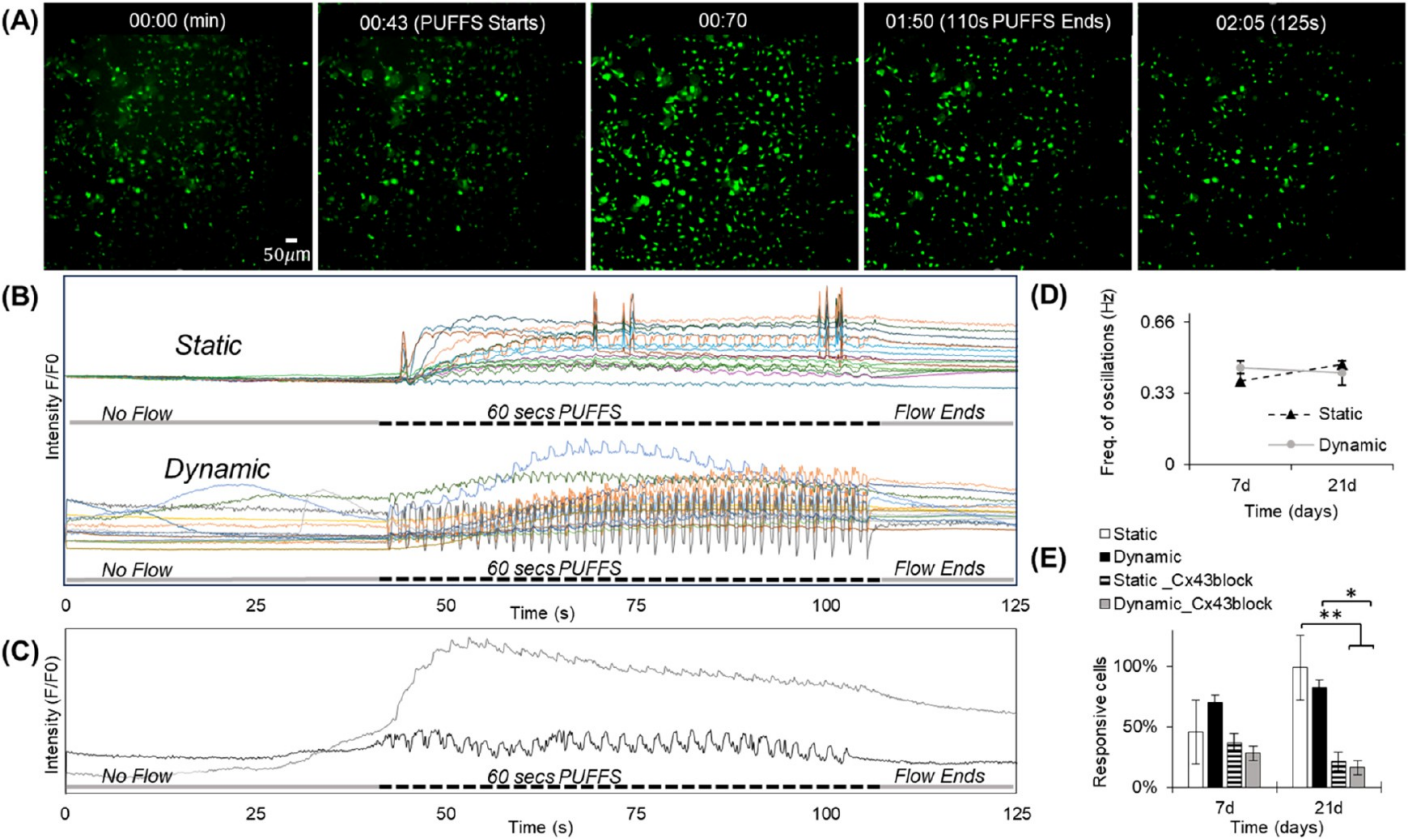
(A) Snapshots of fluorescence images showing the propagation of calcium signaling within MLO-Y4-laden collagen in chamber 2. (B) Representative normalized calcium signaling profiles before, during, and after the application of PUFFS. (C) Plots showing typical single-cell responses. (D) Plot showing frequency of signal oscillations for static and dynamic chips. (E) Number of responsive cells with oscillating signals in the presence and absence of the Cx43 gap junction inhibitor for static and dynamic chips for Days 7 and 21. ** *p* ≤0.010.

The residual Ca^2+^ signaling observed in the presence of GAP26 suggests activity of multiple mechanisms, distinct from Cx43 gap junctions that regulate Ca^2+^ oscillation, including mechanically activated channels Piezo1/2, Trpv channels, and ligand-operated channels, as well as intracellularly mediated Ca^2+^ oscillations. Notably, these signaling mechanisms do not require direct cell-to-cell transmission (e.g., across gap junctions) to initiate calcium influx or to release intracellular stores. Our experiment with the Cx43 selective inhibitor demonstrates that, despite residual signal from these other pathways, gap junctions are a major, perhaps dominant regulator of Ca^2+^ oscillation, particularly at the later time points when functional intracellular junctions are well-established.

### Characterization of Calcium Signaling within Subregions of MLO-Y4-Laden Collagen

We investigated whether regions closer and farther away from PUFFS elicit similar signaling responses. To do so, chamber 2 was divided into three regions: proximal (0–285 μm), central (286–570 μm), and distal (571–855 μm; Figure 9A). First, the number of cells exhibiting oscillatory responses were characterized using *PeakFinder.m* (MATLAB), as explained in the Materials and Methods section (Figures 9B and S14). For Day 3, in the proximal subregion, responsive cells were 21 ± 0.07% (static) and 28 ± 0.08% (dynamic), the central subregion showed 24 ± 0.16% (static) and 21 ± 0.13% (dynamic) of responsive cells, and for the distal subregion, 12 ± 0.14% (static) and 12 ± 0.06% (dynamic) were recorded. For Day 7, in the proximal subregion, responsive cells were 14 ± 0.12% (static) and 19 ± 0.02% (dynamic), the central subregion showed 16 ± 0.15% (static) and 25 ± 0.11% (dynamic) of responsive cells, and for the distal subregion, 16 ± 0.05% (static) and 27 ± 0.15% (dynamic) were recorded. For Day 21, in the proximal subregion, responsive cells were 29 ± 0.05% (static) and 30 ± 0.16% (dynamic), the central subregion showed 31 ± 0.09% (static) and 32 ± 0.13% (dynamic) responsive cells, and for the distal subregion, 40 ± 0.05% (static) and 21 ± 0.04% (dynamic) were recorded. To assess how the signaling properties adapt to PUFFS, we applied multiple rounds of Fluo-4 AM calcium dye staining on the same sample; however, this resulted in significant cell death. As a result, we tried to compare calcium signaling characteristics from independent chips (samples) using calcium dye staining as an endpoint assay. Thus, chips were analyzed for each time point (Days 3, 7, and 21), and their signaling properties were compared. First, single cells in each subregion exhibiting oscillatory calcium signals during PUFFS were pooled together to obtain a cumulative signal that could represent the proximal, central, and distal subregions. To extract a representative calcium signal from each of these subregions, the xcorr function (“signal/SignalSimilaritiesExample”) (MATLAB) was used to cross-correlate calcium signals from all single cells located within each subregion. The xcorr function measures the similarity between two signals at a specified time length and computes the lag differences, where zero lag indicates matching signals. The Excel SORT function was then used to reorder responses based on the distance from PUFFS and group cells with similar responses together. Only cells exhibiting oscillatory responses were analyzed, with 80–400 cells per region obtained from 3 independent chips. Oscillatory cells from proximal, central, and distal groups were averaged into a single response; thus, each chip had 3 regional responses (Figure 9C). For each regional response, the start and stop of PUFFS are indicated by arrowheads. Despite maintaining consistency in threshold settings for all chips and analyzing many single-cell responses (80–400), we found that calcium signals show large variations in regional responses, making direct comparison between chips challenging. We also conducted this experiment in the presence of GAP26 (Cx43 inhibitor) to check if signaling properties are affected; however, due to large signal variations between chips, a direct comparison was not possible.

**Figure 9:**
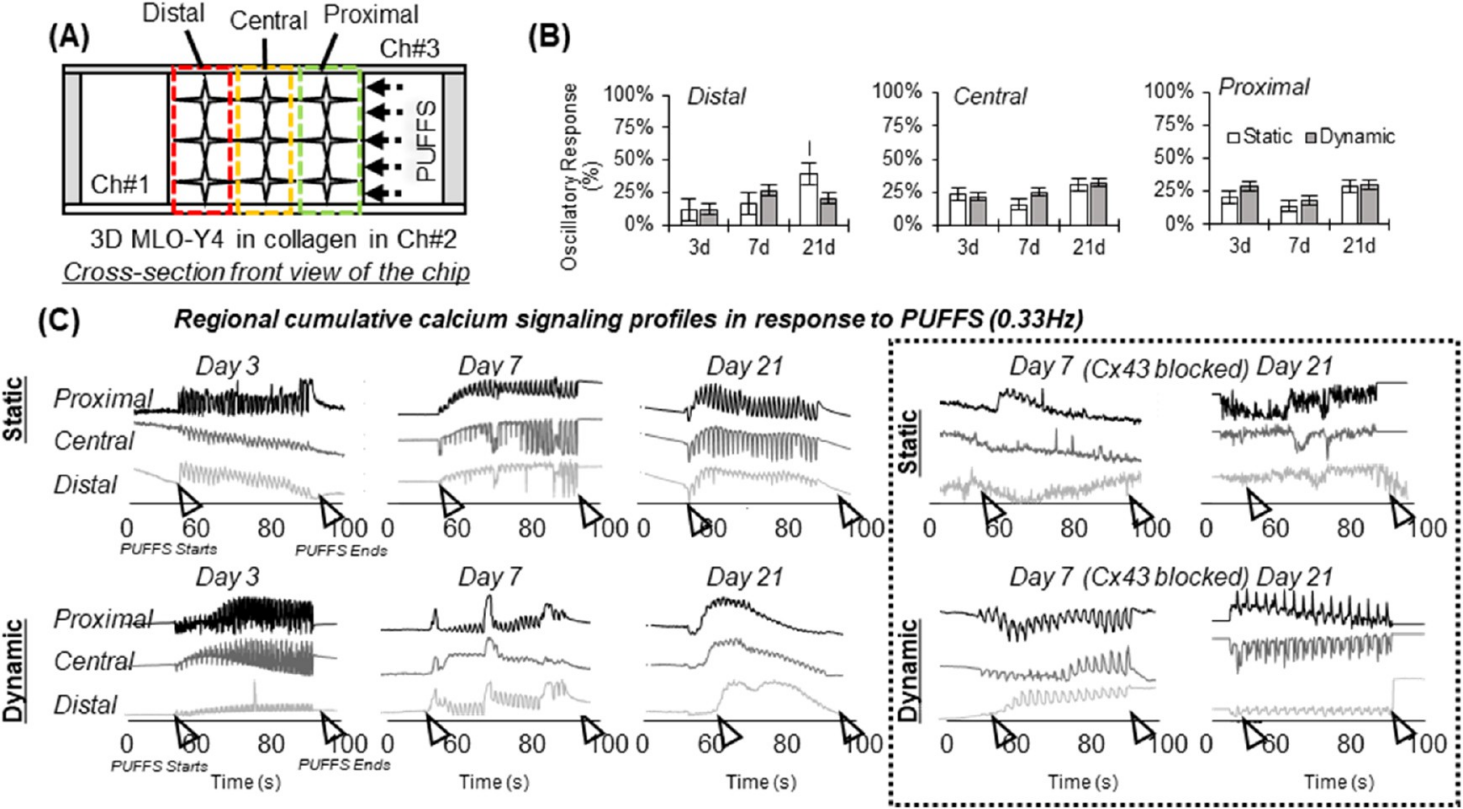
(A) Schematic showing proximal, central, and distal subregions with respect to PUFFS. (B) Plots showing the number of MLO-Y4s with oscillatory responses within each subregion for Days 3, 7, and 21. (C) Left: Representative signaling profiles from various subregions before, during, and after PUFFS. Right: Representative plots when the gap junction was blocked on Day 7. Each signal is captured from an independent chip with application of PUFFS for 60 s (marked by black arrowheads).

In another study, we tested whether sequential application of PUFFS at 0.33 and 1.66 Hz is possible and whether a stable collagen barrier is retained (Figure 10A,B). Process workflow shows the application of PUFFS to both static control and daily PUFFS groups first at 0.33 Hz, followed by a 15 min rest period before a second application of PUFFS at 1.66 Hz. Results show that osteocytes show a signaling spike frequency lower than the PUFFS frequency of 1.66 Hz (Figure 10C). Day 7 static conditions had 55 ± 0.37% response cells and 61 ± 0.03 for PUFFS (1.66 Hz), which increased to 75 ± 0.41% (static) and 79 ± 0.22% (dynamic) by Day 21. We also carried out identical experiments using the Cx43 gap junction blocker (Figure 10D). Unlike the first PUFFS application at 0.33 Hz, blocking by GAP26 did not greatly reduce the oscillatory responses for dynamic conditions during sequential PUFFS application at 1.66 Hz.

**Figure 10:**
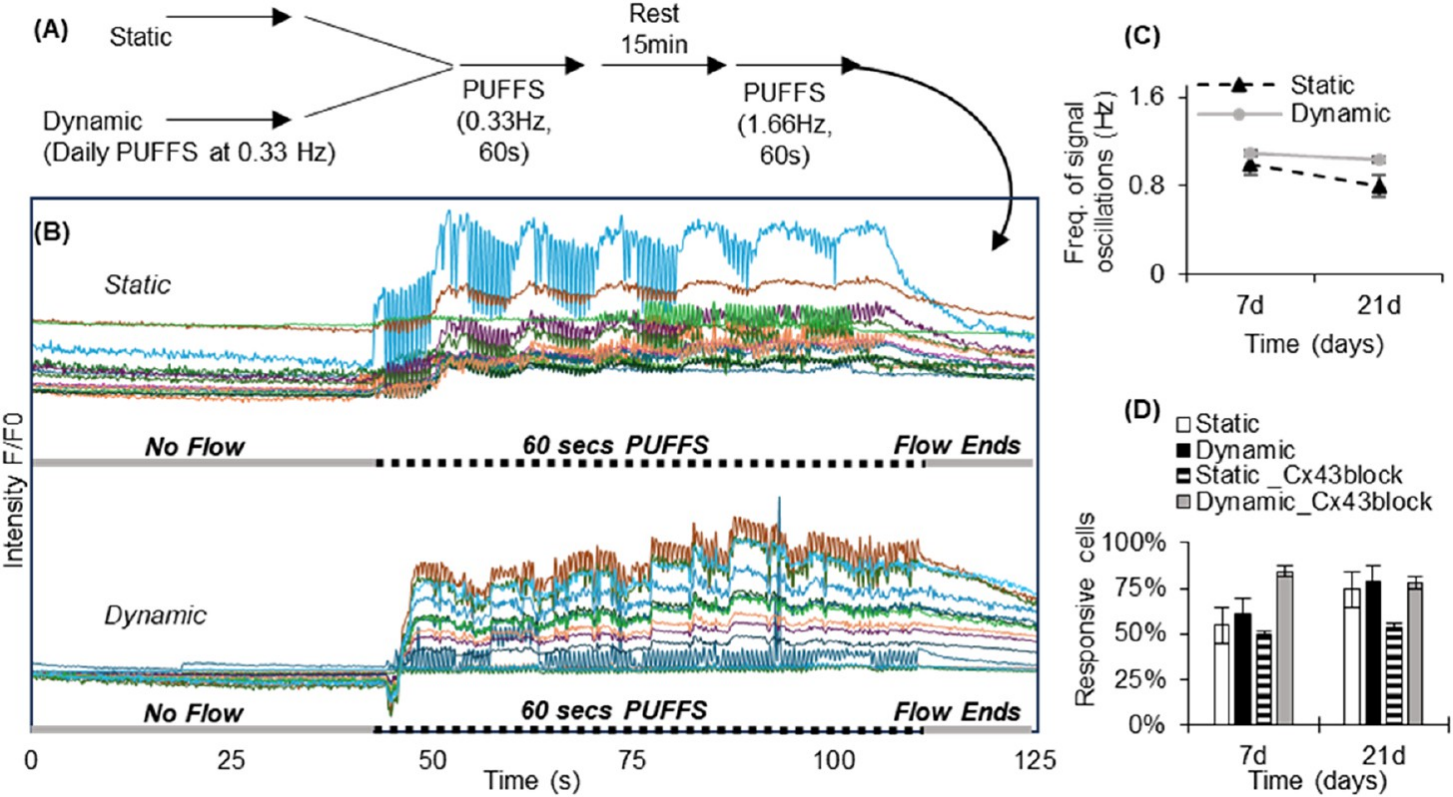
(A) Sequential application of PUFFS at 0.33 Hz (60 s) and then rest for 15 min, followed by PUFFS at 1.66 Hz (60 s). (B) Representative normalized calcium signaling profiles before, during, and after the application of PUFFS. (C) Plot showing frequency of signal oscillations for static and dynamic chips. (D) Number of responsive cells, with oscillating signals, in the presence and absence of the Cx43 gap junction inhibitor for static and dynamic chips for Days 7 and 21 (PUFFS, 1.66 Hz).

Here, we designed and developed a new in vitro model that can be used to study dynamic signaling within a 3D osteocyte network when subjected to defined pulsatile mechanical stimuli. Although aspects of this work have been studied in isolation, our model presents the ability to capture real-time signaling responses under defined mechanical stimulation for long-term 3D osteocyte cultures into an easy-to-use microfluidic platform. For instance, Zhang et al.^99^ used extrusion-printed 3D scaffolds to house human mesenchymal stem cells (hMSCs) and subjected them to defined compressive loading for up to 56 days, while Wilmoth et al.^100^ cultured IDG-SW3 osteocytes within hollow-pillar poly­(ethylene glycol) (PEG) scaffolds for up to 43 days under defined compressive loading conditions. However, neither of the 3D setups allows the study of real-time imaging studies. Another recent work by Yvanoff et al.^37^ showed that MLO-Y4 osteocyte patterns on glass slides can be locally stimulated by AFM probes as well as fluid flow shear stress, and calcium propagation within the osteocyte networks can be investigated; however, this study is restricted to only 2 days.

In this work, we choose to work with immortalized murine osteocyte MLO-Y4, a cell line widely used in the field due to its dendritic morphology, sensitivity to fluid flow and biochemical stimuli, and proven utility in previous publications.^30–37,101,102^ We chose the MLO-Y4 cell line, as it is a well-established model of osteocyte mechanotransduction, and also acknowledged that it is limited in its ability to recapitulate the entire continuum of osteoblast to osteocyte maturation. A series of studies by the Bonewald lab,^103^ who established and characterized the MLO-Y4 cell line who found by the comparison to primary murine osteocytes, that MLO-Y4 recapitulate many of the defining features of the early-to-intermediate stage osteocyte maturation (a) express markers observed in vivo^104^ they are postosteoblastic and do not mineralize their matrix;^105,106^ (b) they display dendritic in morphology;^107^ and (c) establish functional gap junctions^108,109^ and are sensitive to mechanical stimuli^4,106^ and support of both osteoblast^110^ and osteoclast differentiation.^111^ However, the MLO-Y4 line is not suitable for modeling the later stages of osteocyte maturation^112^ and particularly their role as endocrine cells regulating systemic phosphate levels at the kidney through secretion of FGF23. Furthermore, matrix-entombed osteocytes are a postmitotic, terminally differentiated state of the osteogenic lineage in vivo, whereas MLO-Y4 continues to proliferate in vitro, resulting in the high cell densities observed at 21 days in our model. Other available cell lines, such as IDG-SW3, Ocy454, and OmGFP66, have been shown to model a broader range of osteocyte phenotypes, particularly the later stages. However, each of these lines is technically challenging to work with in our model system due to their origin in the Immortomouse background carrying a temperature-sensitive SV40 cassette promoting “immortal” self-renewal crossed with the DMP1-GFP reporter, which conflicts with the green fluorescent calcium reporter used in our studies.

Since the organic portion of bone tissue ECM is mostly collagen (90%), we chose to make 3D osteocyte networks using collagen type I and chose stimuli-evoked calcium (Ca^2+^) transients^113–117^ as a proxy for real-time signaling. We chose PUFFS at 0.33 and 1.66 Hz (15 min/day) based on previous work and found that bone cells respond favorably to repeated short bursts of flow after 10 to 15 min rest periods.^118,119^ A concentration of 2.5 mg/mL was chosen for this study, as it allowed long-term culture of MLO-Y4s under both static and dynamic conditions. Day 21 was chosen as the endpoint, as collagen starts to become unstable beyond this time point due to detachment from the side walls, especially under PUFFS (1.66 Hz). Studies have recorded a variety of responses of osteocytes to fluid flow stimuli.^120–122^ In our studies, when PUFFS was applied, both mechanical deformation of collagen and gap junction-based signaling contributed to calcium signaling profiles. Moreover, the percentage of MLO-Y4s that respond to PUFFS in the form of oscillatory responses (responsive cells) varies with static and dynamic samples and increases the cell number and connectivity by Day 21. In this work, PUFFS-evoked signaling was studied in short-term experiments lasting less than 30 min after adding commercial Fluo-4 AM calcium dye, followed by a semiautomated thresholding method to analyze signaling profiles of 100 s of individual MLO-Y4s. However, due to large variations in calcium signaling profiles, we found it challenging to directly compare results across different chip samples. To address this challenge, we performed Fluo-4 AM staining on the same chip every other day; however, we did not pursue this further due to a decrease in cell viability. In the future, stably transfected variants of MLO-Y4s that express fluorescent fusion proteins labeling the plasma membrane and a genetically encoded calcium indicator could facilitate longitudinal calcium signaling by using the same chip. Due to the large amount of real-time signaling data generated, automated mapping of single cells along with functional connectivity graphs, pairwise coactivity (correlations), and cooccurrences (signal synchrony) should be used in the future. The numerical simulation used in this work also has some limitations. The simulations used a planar representation of the experimental design, which does not fully capture the 3D nature of the actual system. Additionally, the material properties used in the simulation, such as the viscosity and density of the collagen gel, were based on literature reviews and may not accurately reflect realistic variations in collagen. Despite these limitations, our work designed and developed a new chip to enable the study of changes in 3D osteocyte networks subjected to PUFFS. We envision that this minimally invasive chip can be potentially extended to other cell types and could be used to test a range of biophysical and biochemical stimuli.

## Materials and Methods

### Design and Fabrication of Multichambered Microfluidic PDMS Chips Using 3D-Printed Molds

Molds were printed using a prepolymer solution consisting of 40 mL of poly­(ethylene glycol) diacrylate (PEGDA, average *M*_n_ 250) and 0.25% of photoinitiation agent (Irgacure 819, Sigma-Aldrich), 0.01% of TEMPO (Sigma-Aldrich), and 0.5% of 2-isopropylthioxanthone (ITX, Tokyo Chemical Industry). The final prepolymer solution was protected from light with aluminum foil and thoroughly mixed using a vortex for 10 min. Glass slides (25 × 75 × 1 mm, Fisher brand) were precleaned with piranha solution (H_2_SO_4_ and H_2_O_2_; 7:3, stirred for 30 min at 125 rev/min), washed with ethanol and water until reaching a neutral pH, and dried in vacuum oven at 65 °C. The glass slide surface was further modified and stirred (125 rev/min at 50 °C) in 3-(trimethoxysilyl)­propyl methacrylate (TMSPMA; Sigma-Aldrich) and toluene (Sigma-Aldrich) (9:1) and then dried in vacuum at 65 °C. The modified glass slide was sliced using a carbide-metal etching pen into 4 pieces, and each glass piece was adhered to an aluminum print block using a double-sided tape and screwed into the printer stage head. CAD files of the PEGDA master molds were generated using Fusion 360, exported as stl files, then imported into MATLAB to develop sliced image files for the master mold, and exported as PNG files (i.e., black and white resolution 1080 × 1080). Image slices were uploaded as virtual masks into the digital micromirror device (DMD) software controlled by the LabVIEW code. For 3D printing, we utilized a Digital Light Projection (DLP, development kit 1080p 9500 UV, Texas Instruments) platform designed and custom-built by the Soman group.^102,123^ Optical settings consisted of a rotating diffuser to minimize light speckles, a z-stage (25 mm Compact Motorized Translation Stage, ThorLabs), and an ultraviolet light (400 nm) laser source (iBEAM SMART 405, Toptica Photonics). This setup was used to print 250 μm thick PEGDA master molds onto the treated glass slides. The height of the molds was measured with a digital caliper (Mitutoyo). Multiple master molds were printed with minimal batch variation and were used to make chips using replica-casting. The polydimethylsiloxane (PDMS) elastomer was mixed with a curing agent (Sylgard 184, Dow Corning Silicone Elastomer) at 10:1 for 10 min and degassed in a desiccator to remove air bubbles. The PDMS precursor was cast on at least 5 PEGDA master molds evenly spread in a 100 × 15 Petri dish and kept in a vacuum oven at 70 °C overnight with a vacuum shut off to avoid bubble accumulation. After being cooled, the PDMS reverse molds were peeled from the PEGDA master molds. A 2 mm biopsy punch was used to create 6 holes for the inlet/outlet ports. The edges of the PDMS molds were cut, bonded to glass coverslips (22 mm × 22 mm × 0.17 mm, PCS-1.5-2222, Mattek), and precleaned using an overnight acid wash (30% Hydrochloric acid). Prior to bonding, PDMS molds and glass coverslips were plasma-treated (PE-50 model, Plasma Etch Inc.) and heated on a hot plate at 150 °C for an hour. Before use, the chips were sterilized by incubating in 100% ethanol, followed by overnight exposure to UV radiation in a BSL-2 cell culture hood.

### 3D Osteocyte Culture within Three-Chambered Microfluidic PDMS Chips

Before the incorporation of MLO-Y4 cells in the chips, the chips were surface-modified using an established protocol. Briefly, a 2 mg/mL polydopamine (PD, Sigma-Aldrich, H8502) solution was pipetted in chamber 2, incubated at room temperature for 24 h, and then washed with PBS (3×). Then, chip surfaces were incubated in 0.01% poly-l-lysine solution (PL, Sigma-Aldrich, P4707) for 15 min at room temperature and washed three times with PBS, followed by another coating of 0.15 mg/mL rat tail type 1 collagen (RC) for an hour at room temperature. The central channel was washed three times with PBS, dried, and sterilized under UV radiation for 45 min before incorporating the cell solution in the chip. The MLO-Y4 osteocyte cell line (Kerafast, Inc. Boston, MA), maintained in α-MEM (12571063, Gibco), containing l-glutamine, 1% penicillin/streptomycin, 2.5% fetal bovine serum, and 2.5% calf serum, was cultured in flasks coated with 0.15 mg/mL rat tail type 1 collagen (Advanced Biomatrix) at 37 °C under a humidified atmosphere of 5% CO_2_. Upon reaching 75–90% confluency, the cells were trypsinized (0.25%), resuspended in media, and mixed with collagen solution. To prepare the collagen solution, 1333 μL of bovine type 1 collagen (5225 bovine, Advanced BioMatrix, 6 mg/mL) was mixed with 414 μL of 10× HBSS (no. 14065056, Thermo Fisher) and 236 μL of neutralizing agent (Advanced BioMatrix). For all chips, 10 μL of cell solution (∼18,000 cells per chip) was pipetted in the central chamber (Ch#2) and incubated at 37 °C for 30 min to achieve gelation of collagen with encapsulated MLO-Y4s. Media were pipetted in chambers 1 and 3 (side chambers on either side of the collagen barrier) and replenished daily for Days 3, 7, or 21.

### Rheological Characterization of Collagen Gels (Figure S2)

A TA Instruments Discovery Hybrid Rheometer DHR-3 equipped with a lower Peltier plate and 20 mm cross-hatched upper and lower geometries (TA Instruments, New Castle, DE) was used to assess the mechanical properties of collagen gels. Gel slabs of approximately 1 mm thickness were prepared by thermally cross-linking a 2.5 mg/mL bovine collagen solution at 37 °C for 30 min. Prior to analysis, excess superficial water was removed from the gels via gentle blotting with a Kimwipe. Gel slabs were then placed on the rheometer and manually trimmed to yield 20 mm diameter discs. Samples were allowed to equilibrate at 37 °C for 180 s prior to a frequency sweep experiment from 0.1 to 100 Hz with 2% strain. Storage and loss moduli are reported as mean values measured at 1 Hz. All rheological measurements were performed in triplicate. Collagen hydrogels were characterized in bulk, as chip architecture prevents in situ rheological characterization by mechanical rotational rheometry. Hydrogels within the chip are surrounded on three sides by PDMS and covered by glass, rendering them inaccessible from the outside. To work around this limitation, collagen hydrogels were characterized in bulk in a similar fashion to other published work.^124,125^ Briefly, collagen hydrogels were cast and preswelled, resulting in samples approximately 1 mm in height. Collagen slabs were loaded onto cross-hatched 20 mm lower geometry and trimmed to yield 20 mm diameter × 1 mm thick discs. Upper cross-hatched 20 mm geometry was lowered until contact was made with the surface of the sample and then lowered an additional 200 μm to impart a slight normal force and ensure engagement of the hydrogel with the serrated surface of both the upper and lower geometries, preventing slippage.^126^ Collagen gels were prepared in situ from a free-flowing collagen solution within the confined spaces of chamber #2. Since this solution adopts the shape of the chamber prior to gelation, at the time of cross-linking, a relaxed hydrogel scaffold is formed. Subsequent swelling of confined gels may result in compressive forces due to osmotic pressure and the comparatively high modulus of PDMS and glass bounding walls. In cases of high swelling, confinement is anticipated to alter the microstructure of the gel, as confined gels may exhibit greater intermolecular interactions due to tighter packing and enhanced stiffness or make the gel more susceptible to deformation.^127,128^ However, little swelling was observed in bulk collagen gels prepared for rheological characterization in this study. Our model utilizes a microfluidic device where the gel is adhered to the walls as a result of a protein coating that influences the hydrophobic properties of the PDMS and glass surfaces. Therefore, the gel’s integrity is stable within the device, and the exposed surface of the gel at the micropost is most susceptible to alterations. However, this can be assumed negligible at a microscale or for smaller systems.

### Design and Optimization of PUFFS Setup

The overall setup involves two RP peristaltic pumps (#RP-HX01S-1H-DC3VS, Takasago, for PUFFS at 0.33 Hz, and #RP-QX1.5S-1H-DC3 V, Takasago, for PUFFS at 1.66 Hz), a flow controller, and a tubing connected to the inlet and outlets of a chip. During flow stimulation experiments, the settings for PUFFS were adjusted with a controller and monitored at the motor’s frequency reading near 1200 ± 30 Hz (or Pulse Per Second (PPS) by the manufacturer). Based on the manufacturer’s reduction gear ratio (1/50) and 0.015 conversion factors, the actual fluid frequency approximated 0.33 Hz (Figure S11). For the other pump, a similar adjustment was performed to achieve PUFFS at 1.66 Hz. Pressure measurements were performed for 30 min with a relative and differential pressure transmitter (Type 652, Huba control, pressure range: 0–100 kPa). We confirmed 30 kPa as the approximate pressure generated by Takasago’s peristaltic pumps based on pressure measurements collected within 15 min. The pump positive flow tubing was connected to the P1 higher pressure (lower port) of the transmitter. The tubing that administered negative flow (suction) pulled DI water from a Petri dish. We used a G1/8 male to 1/4″ barb fitting at the P1 port, followed by 50 mm of 1/4″ tubing and converted to a 0.8 mm OD barb fitting using a 1/4″ barb to 1/4–28 NPT fitting, a 1/4–28 NPT union and a 1/4–28 NPT to 0.8 mm OD barb. The final 0.8 mm OD barb fitting was attached to the peristaltic pump outlet through 90 mm of 1 mm ID tubing. The pump inlet tubing was submerged in DI water within a Petri dish. Voltage output from the pressure transmitter was monitored with a PD603 Low-Cost OEM Process Meter (Sabre Series). The tubing was filled with DI water, so there was no air in the lines, and the pressure transmitter was left to equalize until it read 0 V while the pump was off. Once zeroed, the pump was turned on and proceeded for 15 min to collect continuous readings. To estimate how long PUFFS takes to enter and exit the channel, we intentionally allowed air to enter the tubing during PUFFS. Using air bubbles, the PUFFS velocity inside chamber 3 was calculated as ∼0.011 m/s. Before applying PUFFS to chamber 3 of the chips, peristaltic pumps were presterilized by perfusing 70% ethanol for 5 min and dried for 15 min. On Day 0, MLO-Y4-laden collagen was cross-linked in chamber 2 of the chips. On Day 1, the media were replenished, and PUFFS was applied for 15 min daily until the target endpoint was reached (Day 3, 7, or 21). All perfusion experiments were performed at room temperature in sterilized biosafety cabinet level 2 (BSL-2). After PUFFS, the media were replenished, and chips were cultured under standard conditions (37 °C, 5% CO_2_).

### Recording of Calcium Signaling and Analysis

PUFFS-evoked calcium signaling within MLO-Y4-laden collagen was tested on Days 3, 7, and 21. For each time point, 3 independent chips were tested. The device stabilizer was generated via Fusion 360, exported as an stl. file and 3D-printed by fused deposition modeling (FDM, Bambu Lab P1P equipped with smooth PEI plate) using poly­(lactic acid) (Figure S1). During printing, the temperature for the nozzle (0.4 mm) and bed plate was adjusted to 250 and 65 °C, respectively. During testing, chips combined with the 3D-printed stabilizer were placed in a 35 × 10 mm Petri dish, and calcium dye solution (500 μL, media +1% PowerLoad +0.1% Fluo-4 AM, #F10489, Thermo Fisher) was pipetted in chambers 1 and 3. Then, chips, covered with aluminum foil, were placed in an incubator (37 °C) for 15 min. Sterile plastic adaptors, created by slicing along the upper marked sections of 1000 μL micropipette tips, were connected at inlets/outlets of the side channels (first and third channels only; Figure 2). Then, fluorescent microscopy (Leica DMI6000 Inverted, 10× objective) was used to record changes in calcium signaling intensities within the region of interest (ROI). Here, the ROI was chosen to be a plane at ∼100 μm from the bottom glass coverslip (approximate center plane of ∼250 μm thick MLO-Y4-laden collagen in chamber 2). Before testing, the pump is connected to the chips’ inlet and outlets and left undisturbed for 15 min. For each calcium signaling experiment, data were captured at least 40 s before PUFFS was applied for 60 s at 0.33 Hz (or 1.66 Hz), and imaging continued for another ∼30 s after the end of PUFFS. Images were captured at a rate of 7 frames per second for a total duration of ∼2.5 min using LASX camera software. For experiments with two PUFFS applications, chips were subjected to PUFFS (0.33 Hz), followed by a rest period of 10–15 min before applying PUFFS at 1.66 Hz. This procedure was followed for both static and dynamic samples. For blocking experiments, 0.5 mg/mL GAP26 (A1044, APExBIO, Cx43 gap junction blocker) was pipetted in chambers 1 and 3 for 45 min and washed 3× with media, before performing signaling experiments. All signal recordings were imported as LOF/LIF files into ImageJ. The measure function and the line tool were used to identify three subregions in chamber #2 based on the distance from PUFFS (Ch#3): proximal (0–285 μm), central (570 μm), and distal (855 μm). For all recorded time frame stacks, a *z*-projection mask with an outline of displacement of individual cells and intensity within the masks were autotraced using a specified threshold within the ROI manager. To minimize motion artifact during image analysis for a particular time-lapse image stack (Figure S13), signal intensities of osteocytes in the frames were used to identify the maximum positive displacement (in the direction of PUFFS shown in c, green) and negative displacement (opposite direction to PUFFS shown in b, blue), and outlines were generated (shown in yellow). ImageJ (FIJI) was used to track the changes in fluorescence intensities within the outlines for all individual frames by subtracting any background artifacts (or scattered fluorescence due to motion blur) using the Corrected Total Cell Fluorescence (CTCF) formula. (d) The image with superimposed displacement images shows the total displacement during PUFFS. A typical maximum displacement of ∼16 μm was noted for our experiments. Using nonflorescence regions (with no cells) as background, the multimeasure option was used to analyze intensities within outlined regions for each time frame slice, and data (mean, area, integrated density) were recorded in a CSV file spreadsheet.

Then, the following procedure was used to identify and analyze “responsive cells” or cells that exhibit an oscillatory response using data collected during PUFFS (60 s). Manual validation of data was used to select the minimum peak prominence threshold (*F*/*F*_0_) of 0.02 (corresponding to the amplitude of the signal). This ensures that the algorithm does not get noise from signals, often associated with the maxima or minima of the signal. This process was applied to all data analyses for all three replicates. Additionally, the number of peaks for each condition were also calculated, as depicted in Figure S14. First, for a particular time-lapse z-stack image set, changes in the fluorescence intensities of single cells were extracted in the form of a spreadsheet; here, Ocy1, Ocy2, ..., Ocy­(*n*) represents “*n*” cells. Then, the MATLAB findpeaks function is used to identify the number of peaks. Using peak prominence criteria of 0.02, (i) for 0.33 Hz PUFFS, 19 peaks were detected; this corresponds to a frequency of 19/60 s = 0.316 Hz, and (ii) for 1.66 Hz PUFFS, 90 peaks were detected; this corresponds to a frequency of 90/60 s = 1.5 Hz. Each response or column was sorted using the filter function, leaving oscillatory responses as the only responses in the spreadsheet. The SORT function was also used to reorder responses based on the distance, grouping cells together. Oscillatory cells within the same distance group were averaged into a single response; thus, each chip had 3 responses that correspond to each subregion (proximal, central, distal). Individual intensities for each chip were plotted over time and *signalcharacteristics.m* script was used to compute the signaling frequency.

### RNA Harvest and RT-qPCR

PCR was performed with unstained replica sample batches. To assess the impact of PUFFS stimulation on gene expression by osteocytes, devices were subjected to dynamic or static conditions for 7 or 21 days. At the end of the study, the central channel containing the stimulated osteocytes was removed, snap frozen, and stored at −80 °C prior to RNA isolation. To isolate RNA, the isolated channels were homogenized in Trizol reagent (Thermo Fisher, Grand Island, NY) using a Precellys bead mill with the MKR28 matrix (Bertin Technologies, Rockville, MD) for three cycles of 30 s at max speed. The RNA was extracted using the recommended protocol and further purified using RNEasyPlus columns (Qiagen Inc., Valencia, CA). The integrity of extracted RNA was verified by formaldehyde–agarose electrophoresis (28S:18S rRNA > 2:1), and RNA purity and quantity were assessed by UV spectrophotometry. The isolated RNA (35 ng/sample) was then reverse-transcribed to cDNA (Quantitect Reverse Transcription Kit, Qiagen) and amplified with (Quantitect SybrGreen PCR Kit, Qiagen) and oligonucleotide primers (Table S2; Azenta Life Sciences) on an Eppendorf Realplex2 instrument. A cDNA library prepared from mouse tibial bone tissue was used to verify primer specificity and optimize reaction efficiency. Dissociation curve analysis was used to verify reaction specificity for each reaction. Following qPCR, expression data were normalized to the geometric mean expression of 2 housekeeping genes (*Gapdh* and *Hsp90ab*). Data are presented as mean fold difference ± 1SD (*n* = 3–5 per group), relative to 7-day static controls, as calculated by the −2^DDCT^ method. Statistical significance of differences between treatment groups was determined by 2-way ANOVA with time (7 days vs 21 days) and treatment (Static/PUFFS) taken as covariates; the Sidak post hoc test was used to evaluate pairwise differences between groups using GraphPad Prism Version 10.4.0 (527).

### Cell Viability, Morphology, Connectivity Plots, and Immunofluorescence Staining

Chips were stained with 0.05% calcein AM and 0.1% ethidium homodimer-1 and washed with PBS before imaging using a Leica DMI6000 Inverted Microscope. For morphology assessment, chips were stained using 1 μg/mL DAPI (diamidino-2-phenylindole) for the nucleus and 1:200 PBS-diluted Alexa Fluor Plus 488 Phalloidin (Thermo Fisher) for f-actin and imaged using an upright Leica DM6 B fluorescent microscope equipped with a THUNDER tissue imager and a *z*-axis focal plane. To quantify the cell number and connectivity within 3D MLO-Y4 networks, the following process was used. The 3D object counter in ImageJ was used to count the correct number of round cells for all time points (time sequence images). The number of round cells obtained from the count mask was divided by the total number of cells (originally from the object mask). The 3D analysis tools gave output values for the *x*, *y*, and *z* locations for the cells by using sliced files of the Nuclei-Blue channel. To determine the percentage of interconnectivity between time points, *x* and *y* values for each sample were imported onto a code-embedded Excel spreadsheet using the Gaussian–Euclidean distance equation. Once imported, distance measurements between cells were calculated with the code; here, we assume that if the distance between two cells was ≤ 50 μm, the cells are connected to each other. For immunostaining, at specific time points (Days 3, 7, and 21), chips were fixed (4% formaldehyde in PBS) for 15 min at room temperature, washed three times, followed by permeabilization using 0.2% for 10 min, and washed again three times. Then, chips were incubated with 1% BSA (blocking agent) for 1 h at room temperature and washed three times using PBS (15 min for each wash). Chips were incubated with primary antibodies overnight at 4 °C and then washed three times with PBS (15 min for each wash), followed by incubation with the secondary antibody solution. Primary antibodies: Mouse Monoclonal Connexin 43 antibody (#35-5000, Thermo Fisher) and Rabbit Polyclonal Integrin Alpha V + β3 antibody (#BS-1310R, Thermo Fisher) or Sclerostin antibody (#219331AP, Thermo Fisher) at a dilution of 1:200 in 0.2% BSA, 0.1% Tween, and 0.3% Triton-X 100 (BTT). Secondary antibodies: Alexa Fluor Plus 647 goat anti-mouse IgG secondary antibody (#A32723TR, Thermo Fisher) and Alexa Fluor 594 Goat anti-Rabbit IgG (H+L) Highly Cross-Adsorbed Secondary Antibody (#A32740, Thermo Fisher) at a dilution solution of 1:1000 in BTT. For control experiments, using Day 21 samples, the same procedure was followed in the absence of primary antibodies. The results show little to no nonspecific fluorescence signals.

### Statistical Analysis

One-way and two-way ANOVA/Tukey tests were used to identify the significant differences. For all results, * *p* ≤ 0.05; ** *p* ≤ 0.010; *** *p* ≤ 0.0010; **** *p* ≤ 0.0001 by 2-way ANOVA; the Sidak posthoc test used for pairwise comparisons.

## Supplementary Material









## Notes

CAD schematics of the device stabilizer; rheological measurement of collagen gels; calculation of velocity and stress during application of PUFFS in chamber 3; numerical solution for velocity and pressure profiles of the simulated PUFFS model; tables showing primer sequences and gene expression; control experiment of antibody staining; schematic and picture of the experimental setup; and process flow for image and signal analysis (PDF)
Representative video file showing deformation of collagen gel with encapsulated fluorescent beads in chamber 2 when subjected to PUFFS at 0.33 Hz (captured at 7 frames per second) (Movie S1) (MP4)
Representative video file showing calcium signal propagation (right to left) across 3D MLO-Y4 networks in chamber 2 during PUFFS application at 0.33 Hz (Movie S2) (MP4)
3D reconstruction of 2D *z*-projection generated by ImageJ volume viewer showing osteocyte cell networks within 3D collagen gel on Day 3 (Movie S3) (MP4)
