# Supplementary material for: 3D Osteocyte Networks under Pulsatile Unidirectional Fluid Flow Stimuli (PUFFS)
Source: ACS Biomater Sci Eng. 2025 Oct 2;11(10):6216–33. doi: 10.1021/acsbiomaterials.5c00730 (PMC12522100; doi:10.1021/acsbiomaterials.5c00730)
Supplement: Supplementary file 1 [file ab5c00730_si_001.pdf]

Supplementary Materials for

3D Osteocyte Networks Under Pulsatile Unidirectional Fluid Flow Stimuli (PUFFS)

Anna-Blessing Merife[1], Arun Poudel[1], Angelika Polshikova[1], Zachary J. Geffert[1], Jason A. Horton[2], Mohammad Mehedi Hasan Akash[3], Anupum Pandey[4], and Saikat Basu[3], Daniel Fougnier[1], and Pranav Soman[1#]

[1]Department of Chemical and Biomedical Engineering, L.C. Smith College of Engineering Syracuse University, Syracuse, NY 13244 USA
[2]Department of Neuroscience and Physiology, Alan and Marlene Norton College of Medicine, SUNY Upstate Medical University, Syracuse NY 13210 USA
[3]Department of Mechanical Engineering, South Dakota State University, Brookings, SD 57007, United States.
[4] Department of Mechanical and Aerospace Engineering, L.C. Smith College of Engineering Syracuse University, Syracuse, NY 13244 USA

[#] corresponding author: psoman@syr.edu

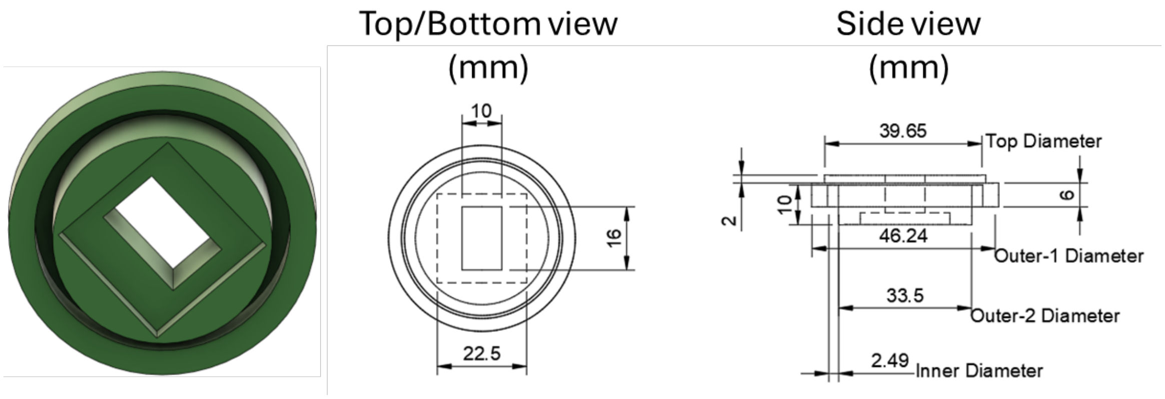

Figure S1. CAD schematic of device stabilizer with top, bottom, and side view dimensions in mm, generated by Fusion 360 drawing tool

Rheological measurement of collagen gels.

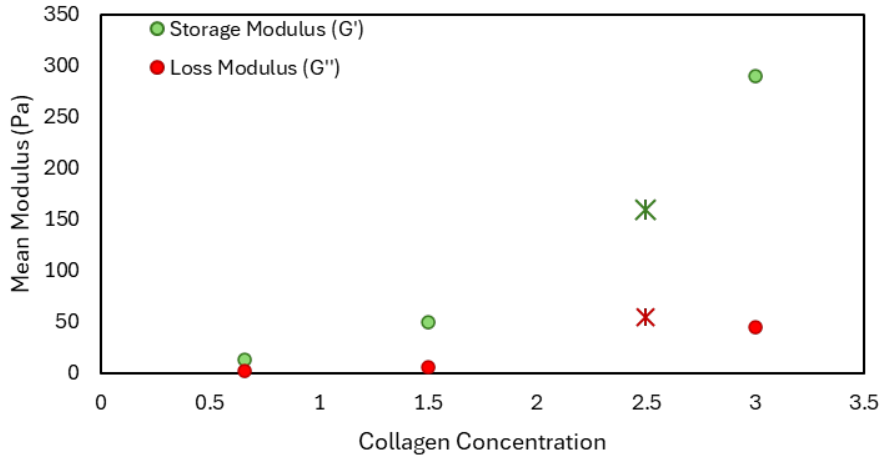

Figure S2. Mean modulus for Storage and Loss plotted for the experimental 2.5 mg/ml with literature values of 0.5, 1.5, and 3 mg/ml

Calculation of velocity and stress during application of PUFFS in chamber 3 of the PDMS microfluidic chip.

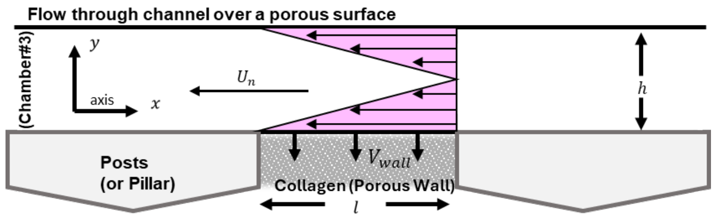

Figure S3. Cartoon schematic of shear stress along the collagen wall (or elastic region) at the gap regions of the channel where x-component velocity travels in the negative direction

Pulsating flow results in oscillating magnitudes of velocity and pressure. In a scenario where PUFFS forms a fluid layer bound by two parallel surfaces, we can determine an analytical solution to solve for

$$x - momentum \qquad \frac{\partial u}{\partial t} = -\frac{1}{\rho}\frac{\partial P}{\partial x} + v\frac{\partial^2 u}{\partial^2 y}$$

velocity that oscillates with time in the x direction. Assume fluid density is constant and no-slip conditions at the wall with boundary conditions $u(y = 0) = 0$ and $u(y = h) = 0$. The continuity equation simplifies to $dv/dy = 0$. The pressure gradient oscillates with time and can be derived as a Fourier series, $\frac{\partial P}{\partial x}(t) = P_x e^{in\omega t}$ where ω is angular frequency for n harmonic. Further derivations will only consider the real part of the imaginary i complex. Thus, the steady-state solution can be represented as , $u(y,\, t) = \emptyset(y)e^{in\omega t}$. We substitute the expressions into the x-momentum equation and rearrange them into second-order differential $in\emptyset e^{inwt} = -\frac{P_x}{\rho}e^{in\omega t} + v\frac{\partial^2\emptyset}{\partial^2 y}e^{in\omega t}$ $\frac{P_x}{\rho v} = y'' - \frac{inw}{v}\emptyset$. We satisfy the conditions of the homogenous equation on the left side to find the complementary solution, and then the particular solution to complete the general solution,

$C_1 e^{\delta_{(n)}y} + C_2 e^{-\delta_{(n)}y}$; $\frac{iP_x}{\rho n} + C_1 \cosh(\delta_n y) + C_2 \sinh(\delta_n y)$ $\quad if\ y\ is\ finite$; where , $\delta = (1 + i)/\sqrt{n\omega/2v}$ and y is finite. Applying the boundary conditions $u(y = 0) = 0$ which gives $C_1 = 0$, and $u(y = h) = 0$ led to the solution for $\emptyset(y)$ that gives the expression for $u(y, t)$, the velocity of PUFFS through chamber #3; $u(y, t) = \frac{iP_x}{\rho_{media} n}\left[1 - \frac{\sinh(\delta_{(n)}y)}{\sinh(\delta_{(n)}h)}\right]e^{in\omega t}$

Collagen can be considered as a porous component. To determine shear stress at the wall, we may assume x-velocity is constant at these regions because the fluid covers the entire collagen surface giving no time for velocity or pressure variations. The continuity equation simplifies to $dv/dy = 0$ and the governing equation becomes $-V_{wall}\frac{\partial u}{\partial y} = \frac{\rho}{\mu}\frac{\partial^2 u}{\partial^2 y}$ where, $\partial p/\partial x = 0$. (16)

By rearranging terms, we can integrate the second-order homogenous differential to find the following general solution: $Ay'' + By' = 0$; $A = \frac{\rho_{media}}{\mu} = v$; $B = V_{wall}$; $u(y) = C_1 e^{-\frac{B}{A}y} + C_2$. The boundary conditions $u(y = 0) = 0$ and $u(y = finite) = 0$ give the exact solution for velocity in the fluid at the collagen region, followed by the shear stress at the wall by derivation $u(y) = U_{(n)}\left[1 - e^{\frac{-V_{wall}y}{v}}\right]$; $\tau_{wall(n)} = \mu\frac{du}{dy} = \mu\frac{V_{wall}U_{(n)}e^{\frac{-V_{wall}y}{v}}}{v}$; Here we can choose a $U_{(n)}$ by plugging known parameters into the oscillating velocity profile (eq.6). The initial pressure ($P_{n=0}$) in the x-direction was determined using Hagen-Poiseuille equation for rectangular channels, where h is width. $\Delta P = \frac{12\mu LQ}{hH^3}$; $P_{n=0} = 36.80\ Pa$; Pressure-driven flow through a porous medium can be calculated using Darcy's Law. We assume that Darcy's velocity is equivalent to the

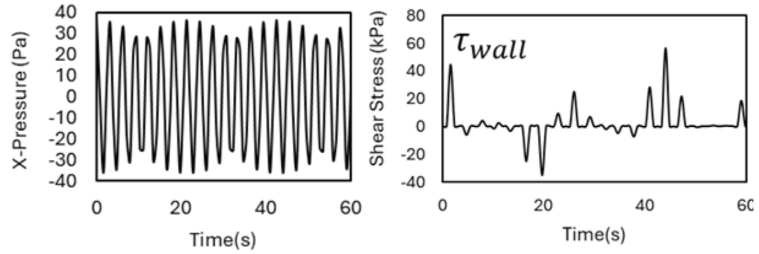

Figure S4. (Left) Plotted oscillating pressure and shear stress (right) along the wall at the collagen surface

constant suction velocity into the collagen wall, which is near $10^{-2} m/s$; $V_{wall} \approx V_{Darcy} = -\frac{k}{\mu} \nabla P = 0.0391 \frac{m}{s}$. Deformation to the collagen wall is perpendicular to the length of the wall. The shear modulus ranged from 61 kPa to 42 kPa, using the following parameters from Table. Finally, to determine the maximum shear stress inflicted along the wall at the collagen surface, we used the computed values of $U_{n=1}$ and V_{wall}. $\tau_{wall(max)} = \mu \frac{du}{dy} = 56.17\ kPa$

Relevant References. I.G. Currie. Pulsating Flow Between Parallel Surfaces. In: Faulkner LL, editor. Fundamental Mechanics of Fluids. Third, 2003; Recktenwald, Steffen M., Christian Wagner, and Thomas John. "Optimizing pressure-driven pulsatile flows in microfluidic devices." Lab on a Chip 21, no. 13 (2021): 2605-2613.; Pérez-Rodríguez S, Huang SA, Borau C, García-Aznar JM, Polacheck WJ. Microfluidic model of monocyte extravasation reveals the role of hemodynamics and subendothelial matrix mechanics in regulating endothelial integrity. Biomicrofluidics 2021;15

Table 1 Parameters used for analytical solution of velocity and pressure profiles in the x-direction

Parameters	Notation	Value	Units
Length of collagen wall	l	6.50E-05	m
Length of channel in ROI	L	2.80E-04	m
Height in y	$h\ (width)$	5.13E-04	m
Height in z	H	2.80E-04	m
Cross-sectional area	A	1.44E-07	m^2
Viscosity media	μ	8.89E-04	Pa/s
Viscosity collagen	μ	1.425	kg/ms
Density (media)	ρ_{media}	1000	kg/m^3
Permeability (collagen)[4]	k	1E-07	m/s
kinematic viscosity (media)	v	8.89E-07	m^2/s
Omega	ω	6.28	radian
Velocity flowrate (pump)	V	0.12	m/s
Oscillating Fluid Velocity wall,	$U_{(n=1)}$	0.018	m/s
Velocity flowrate at wall,	V_{wall}	0.039	m/s
Volumetric flowrate (pump)	Q	1.67E-08	m^3/s

Stress in a collagen layer due to capillary traction on the surface

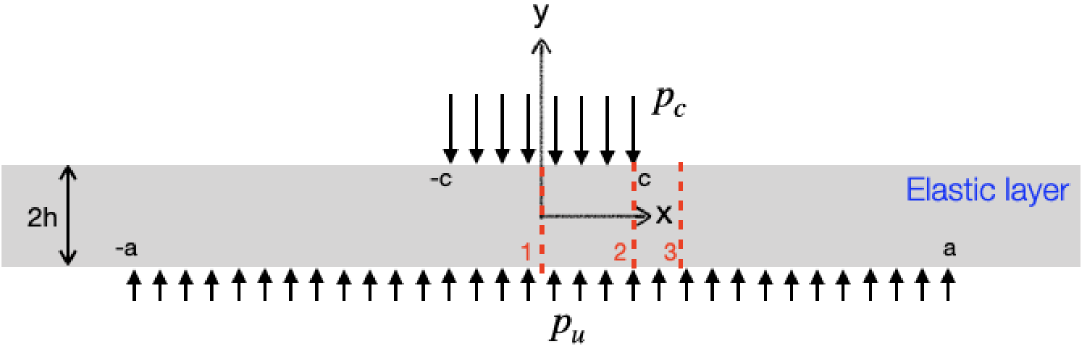

Figure S5: Geometry of the problem studied here; an elastic layer of thickness $2h$ is subjected to a capillary pressure $p_c = \gamma/R$ on the top over a width of $2c$. A uniform pressure of p_u is acting on the bottom surface of the layer. Normal stress (σ_{yy}) at the three sections (marked by the three red dashed lines) are shown in the figure.

Here we calculate the stress in a collagen layer which is subjected to capillary traction due to a steadily propagating droplet. We assume the collagen layer to be purely elastic, and the geometry is sketched in figure S5. We consider the problem to be plain strain problem and the layer thickness ($2h$) to be much smaller than the lateral dimensions. On the top surface a capillary pressure is exerted from the moving droplet. We focus on the static problem with the capillary pressure on top to be stationary. The bottom surface of the elastic layer is exposed to an uniform pressure.
Thus equilibrium requires that,

$$\int_{-c}^{c} p_c(x)\,\mathrm{d}x = \int_{-a}^{a} p_u(x)\,\mathrm{d}x. \tag{1}$$

Since, $p_c = \gamma/R$, the above condition gives that $p_u = p_c(c/a) = \gamma/R(c/a)$. The equilibrium of of the elastic layer is given by,

$$\nabla \cdot \sigma = 0, \tag{2}$$

where σ is the stress tensor which has three independent components of σ_{xx}, σ_{yy}, and σ_{xy}. Equation (2) is solved along with the boundary conditions, $\sigma_{yy}(y = h) = -p_c$, $\sigma_{yy}(y = -h) = -p_u$, $\sigma_{xy}(y = \pm h) = 0$. We follow a Airy's stress function formulation to solve the above equation. In this method, a scalar stress function, Φ is chosen such that, $\sigma_{xx} = \partial^2\Phi/\partial y^2$, $\sigma_{yy} = \partial^2\Phi/\partial x^2$, and $\sigma_{xy} = -\partial^2\Phi/\partial x\partial y$. This choice ensures that Φ identically satisfies eq. (2). However, we are left with the unknown, Φ which is found from the compatibility equation which is the biharmonic equation

$$\frac{\partial^4\Phi}{\partial x^4} + 2\frac{\partial^4\Phi}{\partial x^2\partial y^2} + \frac{\partial^4\Phi}{\partial y^4} = 0. \tag{3}$$

We solve the above equation using Fourier transform, which essentially transform the above PDE to an ODE, $\hat{\Phi}(y;\beta) = \sqrt{\frac{2}{\pi}}\int_0^\infty \Phi(x,y)\cos\beta x\,\mathrm{d}x$,

$$\beta^4\hat{\Phi} - 2\beta^2\hat{\Phi}'' + \hat{\Phi}'''' = 0. \tag{4}$$

Here β acts as a parameter, and derivatives are takes with respect to y. It is to be noted that we have already taken into account the symmetry of the problem about y-axis in defining the Fourier transform using a cosine kernel. Eq. (4) has a general solution

$$\hat{\Phi} = (A + C\beta y)\cosh\beta y + (B + D\beta y)\sinh\beta y, \tag{5}$$

where A, B, C, and D are constants which are found from the boundary conditions. Thus we transform the boundary conditions above in the Fourier domain to find,

$$-\beta^2\Phi[h] = -p_c\frac{\sin\beta c}{\beta},\ -\beta^2\Phi[-h] = -p_c\frac{c}{a}\frac{\sin\beta a}{\beta},\ \Phi'[h] = \Phi'[-h] = 0. \tag{6}$$

Using these boundary conditions, we find the four constants as

$$A = p_c\frac{(c\sin a\beta + a\sin c\beta)(h\beta\cosh h\beta + \sinh h\beta)}{a\beta^3(2h\beta + \sinh 2h\beta)}, \tag{7}$$

$$B = p_c\frac{(-c\sin a\beta + a\sin c\beta)(\cosh h\beta + h\beta\sinh h\beta)}{a\beta^3(-2h\beta + \sinh 2h\beta)}, \tag{8}$$

$$C = -p_c\cosh h\beta\frac{(-c\sin a\beta + a\sin c\beta)}{a\beta^3(-2h\beta + \sinh 2h\beta)}, \tag{9}$$

$$D = -p_c\sinh h\beta\frac{(c\sin a\beta + a\sin c\beta)}{a\beta^3(2h\beta + \sinh 2h\beta)}. \tag{10}$$

Thus we find an analytical solution of the Airy's stress function in Fourier domain. However, the stress function and subsequently the stress components in terms of x and y coordinates are found by numerically evaluating the inverse transform, $\Phi(x, y) = \sqrt{\frac{2}{\pi}}\int_0^\infty \hat{\Phi}(y;\beta)\cos\beta x\,\mathrm{d}\beta$.

Since the forces acting on the elastic layer is purely compressive, the dominant stress component is the normal stress along y, σ_{yy}. In the following, we show how σ_{yy} varies across the elastic layer. For this purpose, we scale all the lengths by h, and stress by p_c. Figure S6 shows the distribution of σ_{yy}/p_c in the elastic layer around the droplet. This plot is obtained for $c/h = 2$ and $a/h = 10$, and $p_u/p_c = c/a = 1/5$. Note that, the stress is maximum at the top surface, beneath the droplet reaching a value of 1 which is marked by color blue. While outside the droplet, the top surface is stress free as marked by the red color. The orange represents the compressive stress at the bottom surface.

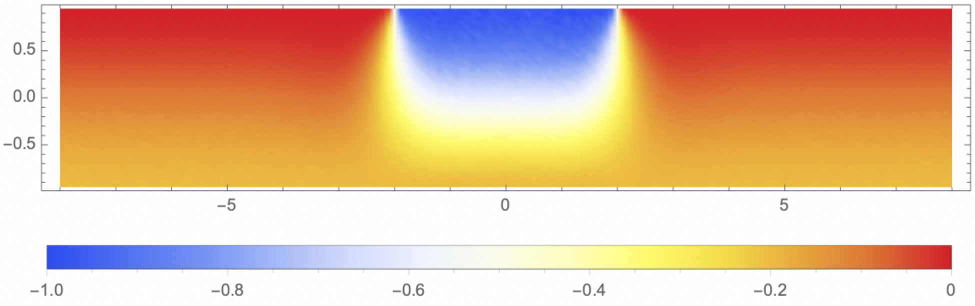

Figure S6: Colormap showing distribution of σ_{yy}/p_c in the collagen layer.

Now we plot the variation of σ_{yy}/p_c with y/h. For this, we consider three cross-sections marked by the red, dashed lines in fig. 1. Figure S7 shows how this dimensionless stress varies along the depth of the elastic layer at the three sections.

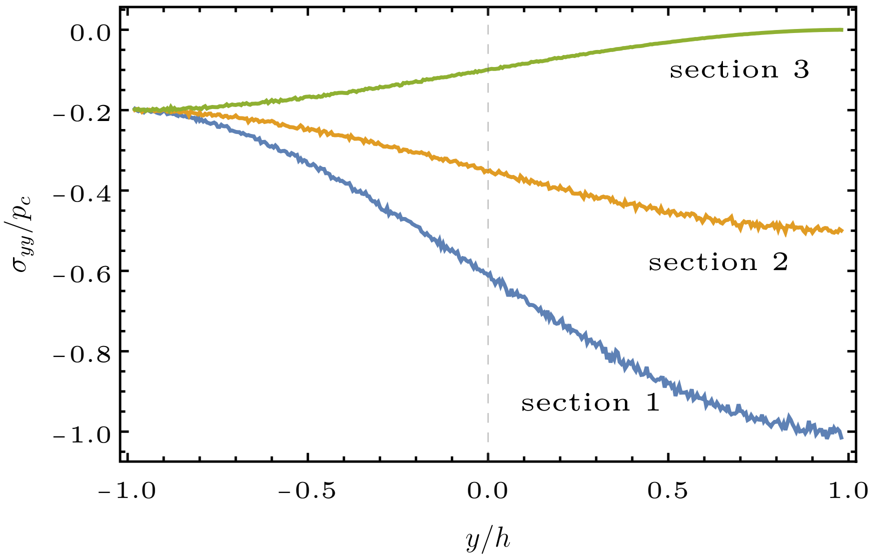

Figure S7: Variation of σ_{yy}/p_c across the thickness of the elastic layer.

Numerically modeled velocity profile in the collagen medium. To study the dynamic interactions between water droplets and a collagen medium, we developed a Eulerian viscous two-phase model to simulate the velocity distribution within the collagen gel, treating water droplets as the primary phase and the collagen gel as the secondary phase. The water droplets were assumed to undergo a combination of rotational and translational motion, and surface tension forces were considered to capture the interactions at the interface between the water and collagen gel phases. The simulation results revealed high-velocity regions near the interface between the water droplets and the collagen gel, with a subsequent decline in velocity within the bulk due to the gel's viscous resistance. By comparing the experimental velocities at specific points within the collagen medium to the simulated values, we identified correction factors that aligned the numerical predictions with the experimental data, thereby validating our simulation model.

Through the in silico architecture shown in **figure S8a**, we have run a two-phase flow simulation to numerically model the velocity field response in the collagen medium (**see figure S9b**) owing to the interfacial interactions with the water drops moving through the side channel (labeled as chamber 1 in figure 1a). The velocity contour plot in the collagen bulk (marked as chamber 2 in figure 1a) reveals a high-velocity region near the interface between the drop and the collagen gel, at the sharp channels that cap the grooved enclosure for chamber 2. Moving cross-stream (relative to chamber 1), note that the negative sign on the velocity values implying the collagen bulk velocity is directed along the negative y-axis. The maximum velocity at the inlets is approximately $-7.03 \times 10^{-2}\ \mathrm{m/s}$, as shown in the color scale. The water interacts with the collagen gel, creating this higher velocity region near the interface. Subsequently, in the yellow and green regions close to the inlets, the velocities range approximately from -2.30×10^{-2} to $-4.66 \times 10^{-2}\ \mathrm{m/s}$. The velocity magnitude declines progressively as we move further into the bulk owing to the collagen gel's viscous resistance, with the least velocity approximating $-0.53 \times 10^{-2}\ \mathrm{m/s}$. Given the "barriers" in the contour plots, the velocity distribution results can be mathematized using a piece-wise function to describe the y-velocity profile of the collagen gel. We define the velocity V_y as a function of y along the vertical direction. From the observations of the contour plot, we can divide the domain into three major regions, namely:

- Inlet region: High-velocity region at the entry locations.
- Interface region: Region where water interacts with the collagen gel.
- Deceleration region: Region where velocity decreases owing to viscous resistance.

Assuming y_1 as the boundary between the inlet and interface regions, y_2 as the boundary between the interface and deceleration regions.

$$v_y(y) = \begin{cases} v_{\max} & \text{if } y \le y_1 \quad (1a) \\ v_{\text{int}}(y) & \text{if } y_1 < y \le y_2 \quad (1b) \\ v_{\text{dec}}(y) & \text{if } y > y_2 \quad (1c) \end{cases}$$

where $v_{\max} \approx -7.03 \times 10^{-2}\ \mathrm{m/s}$ at the inlets, $v_{\text{int}}(y)$ is the velocity in the interface region, which decreases from $v_{\max}$ to $v_{\text{dec, max}}$ and $v_{\text{dec}}(y)$ is the velocity in the deceleration region, which further decreases to the minimum simulated velocity. For simplicity, assuming a linear decrease in velocity in the interface region.

$$v_{\text{int}}(y) = v_{\max} + \left(\frac{v_{\text{dec, max}} - v_{\max}}{y_2 - y_1}\right)(y - y_1) \quad (2)$$

Where $v_{\text{dec,max}} \approx -2.30 \times 10^{-2}$ m/s. Again, considering a bulk linear decrease in the deceleration region.

$$v_{\text{dec}}(y) = v_{\text{dec,max}} + \left(\frac{v_{\min} - v_{\text{dec,max}}}{y_3 - y_2}\right)(y - y_2) \tag{3}$$

where $v_{\min} \approx -0.53 \times 10^{-2}$ m/s is the minimum velocity observed, and y_3 is the vertical extent of the domain. Combining these equations, we get the piecewise function for $v_y(y)$ as:

$$v_y(y) = \begin{cases} -7.03 \times 10^{-2} & \text{if } y \le y_1 \quad (4a) \\ -7.03 \times 10^{-2} + \left\{\frac{-2.30 \times 10^{-2} - (-7.03 \times 10^{-2})}{y_2 - y_1}\right\}(y - y_1) & \text{if } y_1 < y \le y_2 \quad (4b) \\ -2.30 \times 10^{-2} + \left\{\frac{(-0.53 \times 10^{-2} - (-2.30 \times 10^{-2})}{y_3 - y_2}\right\}(y - y_2) & \text{if } y > y_2 \quad (4c) \end{cases}$$

The piece-wise functions mathematically summarize the velocity distribution processed in the simulation results.

Correction factor analysis for velocity discrepancies

The above model findings assume the water drops to be undergoing pure rotation through chamber 1 (see methods) and consequently over-estimate the momentum transferred to the collagen gel. In reality, the drop motion is a mix of rotation and translation along with viscoelastic stretching in presence of micro-scale surfactants, and as such a correction factor is essential to compare and analyze the velocity data from the numerical simulations and the experimental observations. The experimental velocities at two specific points (see figure 1b), the first point (#1) and second point (#2), were measured to be 9.91×10^{-5} m/s and 6.17×10^{-5} m/s. These velocities were determined by tracking the vertical displacement over 27 frames, providing an overall measure of the bulk movement at those points. The total displacement was divided by 2 to account for the oscillatory movement in both the positive and negative y-directions, which is assumed to be symmetrical owing to the periodic flow against the collagen wall. Correspondingly, the simulated absolute velocity regions for the two sample points were 1.12×10^{-2} m/s and 0.53×10^{-2} m/s respectively. The ratio of the velocities, projected numerically and observed experimentally, are hence of the same order, $\mathcal{O}(0)$. Be as it may, given the limitation of the model assumptions, we invoke a correction factor to account for the discrepancies in velocity measurements in the collagen medium. By calculating the ratio of experimental to simulated velocities for each point, we obtain the correction factors, respectively for #1 and #2 as:

$$C_1 = \frac{9.91 \times 10^{-5}}{1.12 \times 10^{-2}} \approx 8.85 \times 10^{-3} \tag{5}$$

$$C_2 = \frac{6.17 \times 10^{-5}}{0.53 \times 10^{-2}} \approx 11.64 \times 10^{-3} \tag{6}$$

The correction factors are hence of the same order, justifying the relevance of the numerical findings when the simulated flow field is revised with a consistent correction coefficient applied across the domain. More importantly, the spatial demarcations of the simulated flow barriers in the collagen medium match almost exactly with the experimental trends.

To further refine our study and account for the complex interactions within the collagen medium, we introduce an elasticity model. This model provides a physics-based rationale for the correction

by incorporating the elastic resistance from the gel layers, which the viscous model alone cannot fully capture. Specifically, the elasticity model examines the distribution of stress and the resultant deformation within the collagen layer when subjected to capillary traction forces. By analyzing the stress response in the collagen layer due to capillary forces from a steadily propagating droplet, we can better comprehend the dynamic interactions.

Here we calculate the stress in a collagen layer which is subjected to capillary traction due to a steadily propagating droplet. We assume the collagen layer to be purely elastic, and the geometry is sketched in fig. 3. We consider the problem to be plain strain problem and the layer thickness $(2h)$ to be much smaller than the lateral dimensions. On the top surface a capillary pressure is exerted from the moving droplet. We focus on the static problem with the capillary pressure on top to be stationary. The bottom surface of the elastic layer is exposed to an uniform pressure. Thus equilibrium requires that,

$$\int_{-c}^{c} p_c(x)\mathrm{d}x = \int_{-a}^{a} p_u(x)\mathrm{d}x \tag{9}$$

Since, $p_c = \gamma/R$, the above condition gives that $p_u = p_c(c/a) = \gamma/R(c/a)$. The equilibrium of of the elastic layer is given by,

$$\nabla \cdot \sigma = 0 \tag{10}$$

where σ is the stress tensor which has three independent components of σ_{xx}, σ_{yy}, and σ_{xy}. Equation (8) is solved along with the boundary conditions, $\sigma_{yy}(y = h) = -p_c, \sigma_{yy}(y = -h) = -p_u, \sigma_{xy}(y = \pm h) = 0$. We follow a Airy's stress function formulation to solve the above equation. In this method, a scalar stress function, Φ is chosen such that, $\sigma_{xx} = \partial^2\Phi/\partial y^2, \sigma_{yy} = \partial^2\Phi/\partial x^2$, and $\sigma_{xy} = -\partial^2\Phi/\partial x\,\partial y$. This choice ensures that Φ identically satisfies eq. (8). However, we are left with the unknown, Φ which is found from the compatibility equation which is the biharmonic equation

$$\frac{\partial^4\Phi}{\partial x^4} + 2\frac{\partial^4\Phi}{\partial x^2\,\partial y^2} + \frac{\partial^4\Phi}{\partial y^4} = 0 \tag{11}$$

We solve the above equation using Fourier transform, which essentially transform the above PDE to an ODE, $\hat{\Phi}(y;\beta) = \sqrt{\frac{2}{\pi}}\int_0^\infty \Phi(x,y)\cos\,\beta x\,\mathrm{d}x$,

$$\beta^4\hat{\Phi} - 2\beta^2\hat{\Phi}'' + \hat{\Phi}'''' = 0 \tag{12}$$

Here β acts as a parameter, and derivatives are takes with respect to y. It is to be noted that we have already taken into account the symmetry of the problem about y-axis in defining the Fourier
transform using a cosine kernel. Eq. (10) has a general solution

$$\hat{\Phi} = (A + C\beta y)\cosh\,\beta y + (B + D\beta y)\sinh\,\beta y \tag{13}$$

where A, B, C, and D are constants which are found from the boundary conditions. Thus we transform the boundary conditions above in the Fourier domain to find,

$$-\beta^2\Phi[h] = -p_c\frac{\sin\ \beta c}{\beta}, -\beta^2\Phi[-h] = -p_c\frac{c}{a}\frac{\sin\ \beta a}{\beta}, \Phi'[h] = \Phi'[-h] = 0 \tag{14}$$

Using these boundary conditions, we find the four constants as

$$A = p_c\frac{(c\sin\ a\beta + a\sin\ c\beta)(h\beta\cosh\ h\beta + \sinh\ h\beta)}{a\beta^3(2h\beta + \sinh\ 2h\beta)} \tag{15}$$

$$B = p_c\frac{(-c\sin\ a\beta + a\sin\ c\beta)(\cosh\ h\beta + h\beta\sinh\ h\beta)}{a\beta^3(-2h\beta + \sinh\ 2h\beta)} \tag{16}$$

$$C = -p_c\cosh\ h\beta\frac{(-c\sin\ a\beta + a\sin\ c\beta)}{a\beta^3(-2h\beta + \sinh\ 2h\beta)} \tag{17}$$

$$D = -p_c\sinh\ h\beta\frac{(c\sin\ a\beta + a\sin\ c\beta)}{a\beta^3(2h\beta + \sinh\ 2h\beta)} \tag{18}$$

Thus we find an analytical solution of the Airy's stress function in Fourier domain. However, the stress function and subsequently the stress components in terms of x and y coordinates are found by numerically evaluating the inverse transform, $\Phi(x, y) = \sqrt{\frac{2}{\pi}}\int_0^\infty \hat{\Phi}(y;\beta)\cos\ \beta x\ \mathrm{d}\beta$.

Since the forces acting on the elastic layer is purely compressive, the dominant stress component is the normal stress along y, σ_{yy}. In the following, we show how σ_{yy} varies across the elastic layer. For this purpose, we scale all the lengths by h, and stress by p_c. Figure 4 shows the distribution of σ_{yy}/p_c in the elastic layer around the droplet. This plot is obtained for $c/h = 2$ and $a/h = 10$, and $p_u/p_c = c/a = 1/5$. Note that, the stress is maximum at the top surface, beneath the droplet reaching a value of 1 which is marked by color blue. While outside the droplet, the top surface is stress free as marked by the red color. The orange represents the compressive stress at the bottom surface.

On the limitations in the numerical model for inter-phase interactions

The numerical simulations offer valuable insights into the interactions between water and collagen gel, but several limitations exist. The simulations used a planar representation of the experimental design, which does not fully capture the three-dimensional nature of the actual system. Additionally, the material properties used in the simulation, such as the viscosity and density of the collagen gel, were based on literature reviews and may not accurately reflect realistic variations due to factors like gel concentration and temperature. In the experiment, both air and water were present at the inlets. However, the simulation only considered water, as air was not the primary concern in studying the interaction between water and collagen gel. Moreover, the simulation did not account for the full rotational motion of water drops, requiring a correction factor to align the simulated velocities with the experimental measurements (see results).

Materials and Methods

In silico test geometry and spatial discretization

The two-dimensional structure of the experimental setup is illustrated in figure 1a, comprising three chambers, with chambers 1 and 3 being identical, bearing heights $h = 0.5131\ \mathrm{mm}$, while chamber 2 has a height $H = 0.7980\ \mathrm{mm}$. In figure 1a, the streamwise length $l = 2.065\ \mathrm{mm}$. Water drops with diameters $D = h$ are modeled to be transiting through chamber 1. The geometry in panel (b) was spatially meshed, resulting in 47,740 linear quadratic elements. This design includes seven inlets for water entry, with the entire chamber 2 filled with collagen gel.

Numerical simulation of interfacial mechanics-induced bulk motion in the collagen medium

The interaction of water (phase 1) with the surface of collagen gel (phase 2) through the inlets is modeled as a viscous laminar transient flow with SIMPLEC pressure-velocity coupling and second-order upwind spatial discretization. To replicate this interaction, the Eulerian multiphase model is used between the primary and secondary phases [1]. This Eulerian multiphase model tracks the continuity and momentum for each phase [2]. The simulation begins with the continuity equation [3], which ensures the mass conservation of each phase, mathematically implying

$$\frac{\partial(\alpha_b \rho_b)}{\partial t} + \nabla \cdot (\alpha_b \rho_b \vec{v}_b) = 0 \tag{17}$$

Here $\vec{v}_b$ represents the velocity of phase b, α_b denotes the volume fraction, and ρ_b is the density of phase b. By adhering to equation 7, the simulation ensures that the mass of the water and collagen gel is conserved within the computational domain. This conservation also means that there are no sources or sinks in the system. The surface tension force [4] is considered to model the interaction at the interface between the water and collagen gel phases, and is quantified as

$$F_\sigma = \sigma_b \kappa \nabla \alpha \tag{18}$$

where σ_b is the surface tension coefficient, κ is the curvature of the interface, and $\nabla\alpha$ is the gradient of the volume fraction. The curvature κ[3] of the interface is calculated as:

$$\kappa = -\nabla \cdot \left(\frac{\nabla\alpha}{|\nabla\alpha|}\right) \tag{19}$$

The above equations capture the dynamic behavior of the interface between the water and collagen gel phases. Subsequently, the momentum equation [5] describes the forces acting on each phase. The conservation of momentum principle, which is fundamental for determining the velocity and pressure fields within the system, is mathematized as

$$\frac{\partial(\alpha_b \rho_b \vec{v}_b)}{\partial t} + \nabla \cdot (\alpha_b \rho_b \vec{v}_b \vec{v}_b) = -\alpha_b \nabla p + \nabla \cdot (\alpha_b \overline{\overline{\tau_b}}) + \alpha_b \rho_b \vec{g} + \vec{F}_\sigma \tag{20}$$

In this equation, p represents the pressure shared by both phases, $\overline{\overline{\tau_b}}$ is the stress-strain tensor, and $\vec{g}$ denotes the gravitational acceleration. This comprehensive equation incorporates the effects of pressure gradients, viscous stresses, gravitational forces, and surface tension, providing a detailed description of the flow dynamics within the system. The stress tensor for phase b is formulated to capture the viscous behavior and is given by [6]

$$\overline{\overline{\tau_b}} = \mu_b \left(\nabla \vec{v}_b + \nabla \vec{v}_b^T\right) - \frac{2}{3} \mu_b (\nabla \cdot \vec{v}_b) \vec{I} \tag{21}$$

with μ_b being the dynamic viscosity, $\vec{v}_b$ as the velocity vector, $\nabla\vec{v}_b$ as the velocity gradient tensor, $\nabla\vec{v}_b^T$ as the transpose of the velocity gradient tensor, $\nabla \cdot \vec{v}_b$ as the divergence of the velocity field, and $\vec{I}$ as the identity matrix. The above
equation represents the stress distribution within the system. Finally, the volume fraction [7] for each phase is tracked using the volume fraction equation which monitors how the water phase infiltrates and interacts with the collagen gel, and is given by

$$\frac{\partial \alpha_b}{\partial t} + \nabla \cdot (\alpha_b \vec{v}_b) = 0 \tag{22}$$

The convergence of the numerical solution is determined by minimizing the residuals of the mass and velocity components. For the simulations of pressure-gradient-driven laminar flow, typical execution times range from 0.2 to 0.5 hours for 500 iterations with a time-step of 0.001 seconds, utilizing a 4-processor-based parallel computation setup operating at 3.1 GHz on Xeon nodes. Assuming pure rotational motion of the water drops through chamber 1, the inlet velocity (at the water-collagen interface) was periodically specified by a user-defined function, bearing the following magnitude in the negative y-direction (see figure 1):

$$\mathcal{V}_c = \sqrt{r \times g} = \sqrt{\frac{0.5131}{2} \times 10^{-3} \times 9.81} = 0.0502 \text{ m/s} \tag{23}$$

where r is the radius of the water drop and g is the gravitational acceleration. Physical parameters such as the density and viscosity of water are assumed to be 1000 kg/m^3 and 0.001 kg/(m.s), respectively. For collagen gel, the density is assumed to be 1300 kg/m^3 [8] and viscosity 1.425 kg/(m.s)[9].

Figures and Tables

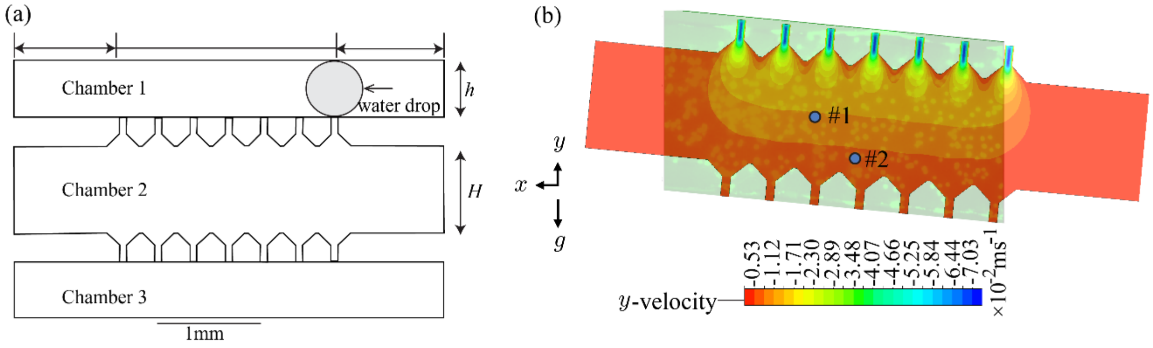

Figure S8. Panel (a) shows the planar numerical domain, extracted as a projection of the experimental setup. Panel (b) compares the experimental and numerical results through superposition, showing two points (#1) and (#2) in the numerical velocity profile used to calculate the correction factor (see results) where the color scale presents the y-component of velocity in units of 10^{-2} m/s, with dark blue indicating higher downward velocities and red indicating lower downward velocities. The dark red sections at the edges indicate places where the vertical velocity approaches zero (no-slip). The negative sign before the velocity indicates the velocity directed towards negative y-axis. The 1-mm scale reference bar is at the bottom of panel (a). Additionally, next to panel (b), x and y axes establish the spatial orientation and g signifies the gravity direction in the simulation.

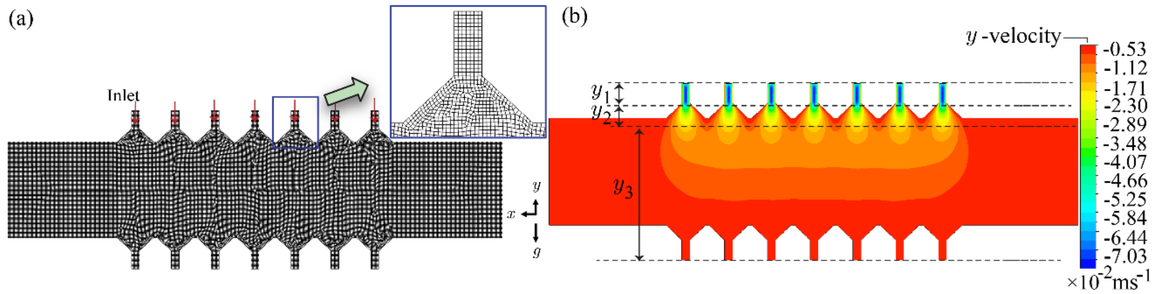

Figure S9. Panel (a) shows the mesh visual and its zoomed-in details, with the enforced inlets marked. Therein the red arrows show the direction of inlet flow. Panel (b) presents the velocity magnitude contours in the collagen gel, where the color scale presents the y-component of velocity in units of 10^{-2} m/s, with dark blue indicating higher downward velocities and red indicating lower downward velocities. The dark red sections at the edges indicate places where the vertical velocity approaches zero (no-slip). The negative sign before the velocity indicates the velocity directed towards negative y-axis. Additionally, next to panel (b), x and y axes establish the spatial orientation and g signifies the gravity direction in the simulation.

Relevant references.

[1] Mohammad Mehedi Hasan Akash, Nilotpal Chakraborty, Jiyan Mohammad, Katie Reindl, and Saikat Basu. Development of a multiphase perfusion model for biomimetic reduced-order dense tumors. Experimental and Computational Multiphase Flow, 5(3):319-329, 2023.

[3] Mamoru Ishii and Takashi Hibiki. Thermo-fluid dynamics of two-phase flow. Springer Science & Business Media, 2010.

[4] Jeremiah U Brackbill, Douglas B Kothe, and Charles Zemach. A continuum method for modeling surface tension. Journal of Computational Physics, 100(2):335-354, 1992.

[5] Seung Jun Lee, Byoung Jae, Ik Kyu Park, and Han Young Yoon. Comparative study of the two-fluid momentum equations for multi-dimensional bubbly flows: Modification of reynolds stress. Journal of Mechanical Science and Technology, 31:207-214, 2017

[6] George Keith Batchelor. An Introduction to Fluid Dynamics. Cambridge University Press, Cambridge, UK, 1967.

[7] Shankar Subramaniam. Lagrangian-eulerian methods for multiphase flows. Progress in Energy and Combustion Science, 39(2-3):215-245, 2013.

[8] Myung Gu Yeo and Geun Hyung Kim. Preparation and characterization of 3d composite scaffolds based on rapid-prototyped pcl/β-tcp struts and electrospun pcl coated with collagen and ha for bone regeneration. Chemistry of materials, 24(5):903-913, 2012.

[9] Advanced BioMatrix. Viscosity of purecol, nutragen, and fibricol collagen. Technical report, Advanced BioMatrix, 2008 .

Table S2. Oligonucleotide Primers.

Genes of interest	Gene Symbol	NCBI-blast Accession	Forward Primer (5'-3')	Reverse Primer (5'-3')	Annealing Temp (ºC)	mRNA target ('5-'3)	Amplicon Size (BP)
alkaline phosphatase, liver/bone/kidney	*Alpl*	NM_007431.3	CCAACTCTTTTGTGCCAGAGA	GGCTACATTGGTGTTGAGCTTTT	60	44-153	110
gap junction protein, alpha 1 (Connexin 43)	*Gja1*	NM_010288	ACAGCGGTTGAGTCAGCTTG	GAGAGATGGGGAAGGACTTGT	60	115-220	106
Podoplanin	*Pdpn*	NM_010329	ACCGTGCCAGTGTTGTTCTG	AGCACCTGTGGTTGTTATTTTGT	60	7-165	159
Sclerostin	*Sost*	NM_024449	AGCCTTCAGGAATGATGCCAC	CTTTGGCGTCATAGGGATGGT	62	81-214	134
Fibroblast Growth Factor 23	*Fgf23*	NM_022657	ATGCTAGGGACCTGCCTTAGA	AGCCAAGCAATGGGGAAGTG	60	1-81	100
Dentin Matrix Protein	*Dmp1*	NM_016779	CATTCTCCTTGTGTTCCTTTGGG	TGTGGTCACTATTTGCCTGTG	60	12-196	185
phosphate regulating endopeptidase homolog, X-linked	*Phex*	NM_011077	GAAAGGGGACCAACCGAGG	AACTTAGGAGACCTTGACTCACT	60	35-139	105
matrix extracellular phosphoglycoprotein with ASARM motif	*Mepe*	NM_053172	GTCTGTTGGACTGCTCCTCTT	CACCGTGGGATCAGGATACA	60	36-165	130
tumour necrosis factor receptor superfamily member 11B (OPG, Osteoprotegerin)	*Tnfrsf11b*	NM_011613	CGCTCTGTTCCTGTACTTTCG	GAGTCCTGCAAATCTGCGTT	55	195-308	114
tumor necrosis factor (ligand) superfamily, member 11 (RANK-L)	*Tnfsf11*	NM_011613	AGCCGAGACTACGGCAAGTA	AAAGTACAGGAACAGAGCGATG	57	13-213	201

Housekeeping genes	Gene Symbol	NCBI-blast Accession	Forward Primer (5'-3')	Reverse Primer (5'-3')	Annealing Temp (ºC)	mRNA target ('5-'3)	Amplicon Size (BP)
glyceraldehyde-3-phosphate dehydrogenase	*Gapdh*	NM_008084	AGGTCGGTGTGAACGGATTTG	TGTAGACCATGTAGTTGAGGTCA	59	8-130	123
heat shock protein 90 alpha (cytosolic), class B member 1	*Hsp90ab1*	NM_008302	GTCCGCCGTGTGTTCATCAT	GCACTTCTTGACGATGTTCTTGC	57	1069-1236	168

Table S3. Relative fold-change of gene expression by RT-qPCR

Av. fold-diff. (StDev)	Early (7d)		Late (7d)	
Gene	Static	Dynamic	Static	Dynamic
Alpl •■▲	1.00b,c<br>(0.18)	-3.73a<br>(2.15)	-26.30a,d<br>(1.88)	-6.48c<br>(1.58)
Gja1 ■	1.00<br>(0.60)	1.23<br>(0.73)	1.99<br>(0.72)	2.27<br>(0.87)
Pdpn ■▲	1.00b,c<br>(0.32)	-3.06a<br>(1.63)	-10.69a,d<br>(1.92)	-5.52c<br>(1.15)
Sost ■	1.00c<br>(0.16)	1.19d<br>(0.77)	-14.94a<br>(1.57)	-13.23b<br>(2.09)
Fgf23 ■	1.00c<br>(0.01)	1.48d<br>(0.34)	-52.79a*<br>(17.00)	-57.39b*<br>(37.46)
Rankl •■▲	1.00b<br>(0.09)	-5.58a,d<br>(1.12)	-0.35<br>(3.66)	1.07b<br>(0.07)
Opg ■▲	1.00c<br>(0.09)	1.04<br>(1.16)	-11.02a,d<br>(4.71)	-3.71c<br>(1.57)
Phex ■	1.00c<br>(0.18)	-1.26<br>(-1.12)	-7.77a<br>(5.90)	-6.04<br>(3.32)
Mepe •■▲	1.00c<br>(0.17)	-1.65d<br>1.46	-15.31a,d<br>(1.36)	-11.60b,c<br>(1.53)
Dmp1 ■▲	1.00b,c<br>(0.19)	1.83a,d<br>(1.34)	-29.31a,d<br>(2.64)	-9.48b,c<br>(1.46)

Two-way ANOVA Significance of factors

Factors		Pairwise Comparisons
•Time		a p<0.05 by 2-way ANOVA vs. 7d Sta
■Stimulus		b p<0.05 by 2-way ANOVA vs. 21d S
▲Time*Stimulus		c p<0.05 by 2-way ANOVA vs. 7d dy
		d p<0.05 by 2-way ANOVA vs. 21d d
		*not reliably detected by qPCR

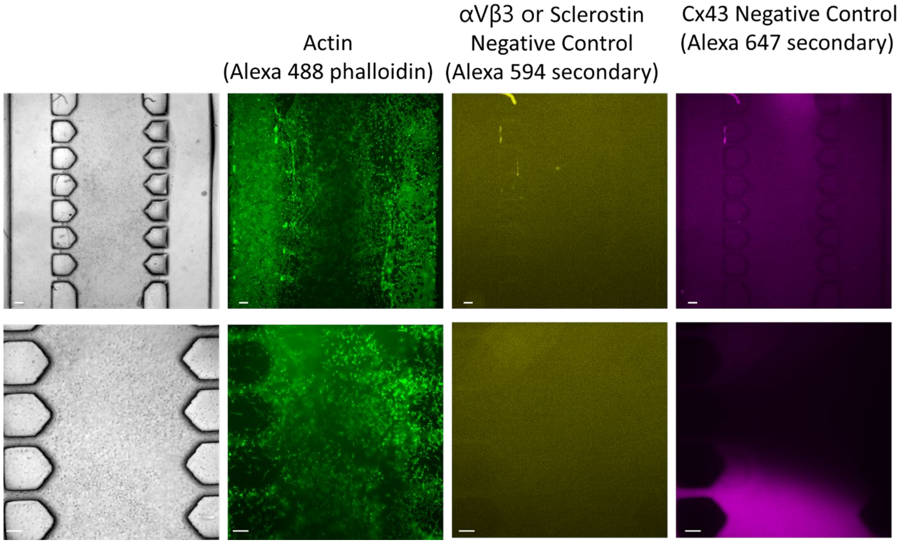

Figure S10. Control experiment of secondary antibody stains to show non-specific binding using Day 21 samples. Brightfield image of the sample to the far left. Actin was imaged under the green channel, Alexa 594 secondary was imaged under the red channel (depicted as a yellow hue signal), and Alexa 647 secondary was imaged under the far-red channel [illegible]

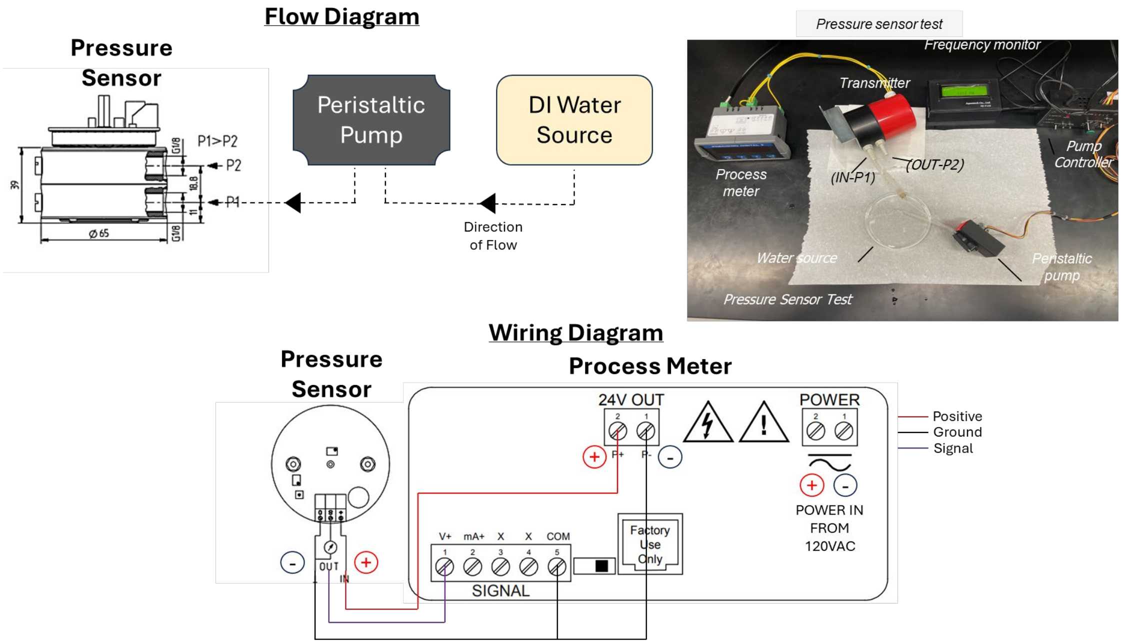

Figure. S11. Schematic of connections, instrument parts, and materials to measure the pressure administered by the peristaltic pumps

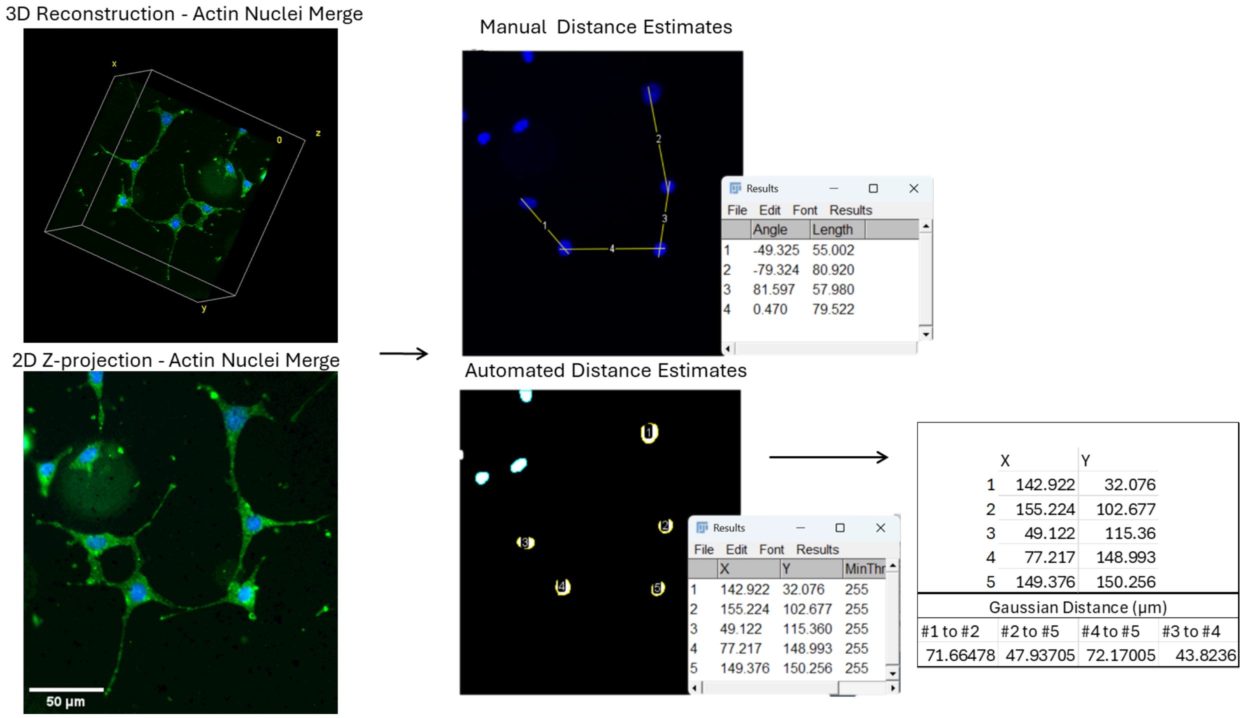

Figure S12. Process flow showing the use of ImageJ (FIJI) to detect the location of cell nuclei and to calculate the distance between neighboring cells using the Gaussian distance formula. Based on this, the distance threshold for connections was set to below 50 µm.

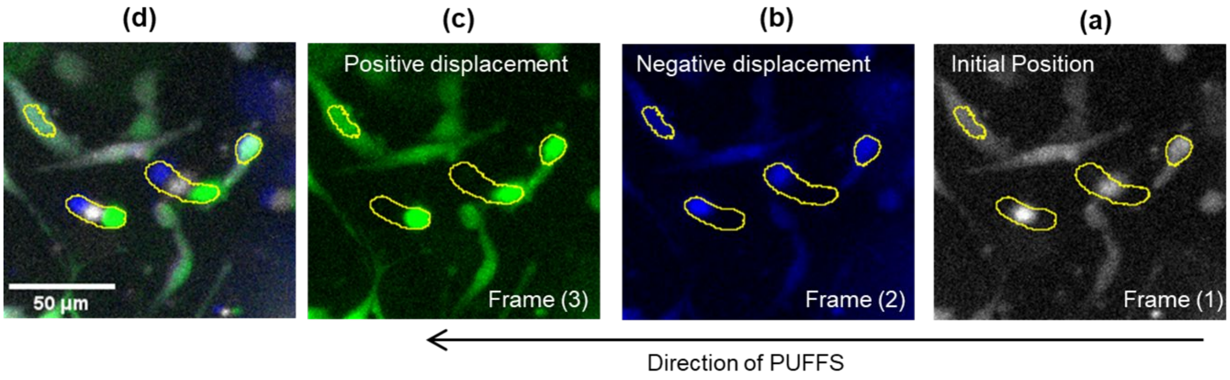

Figure S13: To minimize motion artifact during image analysis for a particular time-lapse image stack, signal intensities of osteocytes in the frames were used to identify the maximum positive displacement (in the direction of PUFFS shown in c, green) and negative displacement (opposite direction to PUFFS shown in b, blue), and outlines was generated (shown in yellow). Image J (FIJI) was used to track the changes in fluorescence intensities within the outlines for all individual frames by subtracting any background artifacts (or scattered fluorescence due to motion blur) using the Corrected Total Cell Fluorescence (CTCF) formula. (d) Image with superimposed displacement images showing the total displacement during PUFFS. A typical maximum displacement of ~16 µm was noted for our experiments.

MATLAB "findpeaks" analyzes and then identifies local maxima of a signal and returns the x,y indexes or values

[PeaksVal, PeaksLoc] = findpeaks(FiltData, time, 'MinPeakProminence', 0.02)

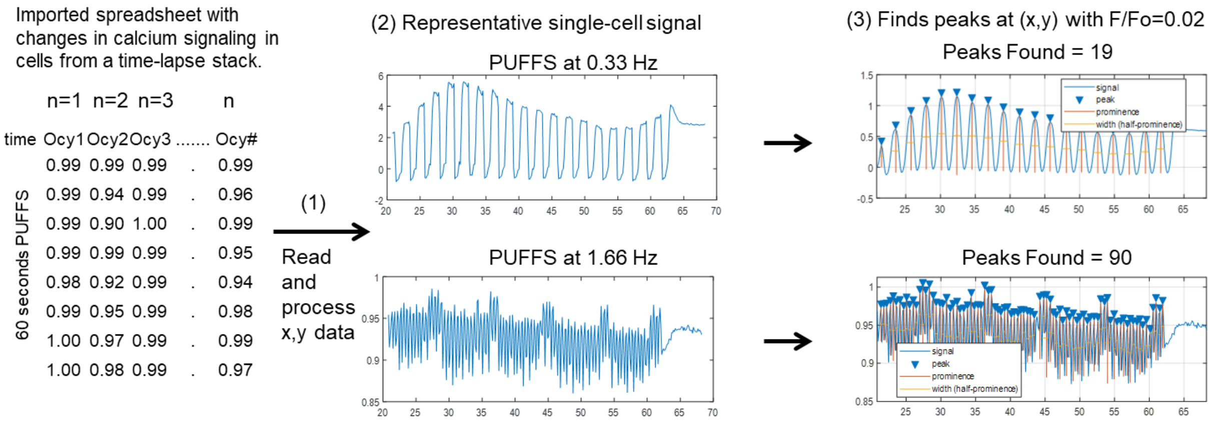

The algorithm repeats to generate the number of peaks for all cells in a particular time-lapse z-stack image set.

Figure S14. During application of PUFFS (60s), cells are identified in a particular time-lapse z-stack and the changes in the fluorescence intensities are extracted in the form of a spreadsheet; here Ocy1, Ocy2 …Ocy(n) represents 'n' cells. Then, MATLAB findpeaks function is used to identify the number of peaks. Manual validation of data was used to select the minimum peak prominence threshold (F/F0) of 0.02 to prevent the algorithm to incorrectly identify amplitude peaks. For this representative data set, for 0.33Hz PUFFS, 19 peaks correspond to a frequency of 19/60s = 0.316Hz, and for 1.66Hz PUFFS, 90 peaks correspond to a frequency of 90/60s = 1.5Hz. Cumulative results are reported in Figures 8D and 10C.

Movie S1. Caption: Representative video file showing deformation of collagen gel with encapsulated fluorescent beads in chamber 2 when subjected to PUFFS at 0.33 Hz. (Captured at 7 frames per second)

Movie S2. Caption: Representative video file showing calcium signal propagation (right to left) across 3D MLO-Y4 networks in chamber 2 during PUFFS application at 0.33 Hz.

Movie S3. Caption: 3D reconstruction of 2D z-projection generated by ImageJ volume viewer showing osteocyte cell networks within 3D collagen gel on Day 3.
